# Barriers and Facilitators to Nurse-Provider Communication in the Emergency Department: A Scoping Review

**DOI:** 10.1177/08445621251320710

**Published:** 2025-03-17

**Authors:** Sylwia Borawski, Jody Ralph, Adam Mulcaster

**Affiliations:** 1 8637 University of Windsor, Windsor, ON, Canada

**Keywords:** Nurse-provider communication, emergency department, communication barriers, communication breakdown, interprofessional collaboration, communication technology

## Abstract

**Background:**

Effective nurse-provider communication in the emergency department (ED) is crucial but often hindered by hierarchical dynamics and high workloads

**Objectives:**

This review aimed to examine, systematically map, and identify gaps in existing literature concerning ED registered nurse-provider communication.

**Eligibility Criteria:**

Studies focused on direct communication between bedside registered nurses (RNs) and providers, including physicians, physician assistants (PAs), and nurse practitioners (NPs) in the ED, encompassing verbal, non-verbal and electronic communication. Non-direct patient care roles were excluded. Sources of Evidence: A search in CINAHL, MEDLINE and ProQuest Nursing & Allied Health identified 1978 sources, of which 37 studies were included: 15 qualitative, 9 quantitative, 4 mixed methods, 6 commentaries, 1 performance improvement project, and 1 scoping review.

**Charting Methods:**

Data were extracted using Joanna Briggs Institute (JBI) guidelines and thematically analyzed according to Levac’s framework.

**Results:**

Four key themes emerged: (i) Interruptions hinder communication but can be reduced by electronic supports facilitating asynchronous communication, (ii) Power imbalances and high workload/communication load impede effective communication, emphasizing the need for structured communication tools and interprofessional communication training, (iii) Shared workspaces, electronic supports, and collaborative, respectful interactions enhance communication (iv) Timely updates and collaborative planning are valued, emphasizing the significance of consistent communication.

**Conclusions:**

This review identified interventions that can improve ED nurse-provider communication, including electronic supports, shared workspaces, structured communication tools, and interprofessional communication training. Future research should evaluate these strategies’ effectiveness and explore regional differences, particularly in Canada, where the literature is limited.

## Background and Purpose

The emergency department (ED) is a rapidly changing, high-stress environment (Abourbih et al., 2015), where registered nurses and providers spend approximately 89% of their working hours communicating (Spencer et al., 2002). Despite frequent communication, there is a lack of ED-specific literature on this topic (Daheshi et al., 2023). Recently, recognition of the role of effective nurse-provider communication in the ED for patient safety and outcomes has grown, leading to several related publications (Abourbih et al., 2015; Amaniyan et al., 2020; Daheshi et al., 2023; Hettinger et al., 2020). Although these propose recommendations to enhance ED communication processes, there is a lack of follow-up on their effectiveness and no consensus on the most appropriate changes. Consequently, a knowledge gap exists surrounding facilitators, barriers, content consensus, and preferred tools.

Although numerous studies on registered nurse-provider communication have been published in other regions, particularly in the United States, the scarcity of Canadian literature on this topic presents difficulties in understanding and addressing the unique dynamics of ED nurse-provider communication in the Canadian context. Differences between the Canadian and American healthcare systems, including staffing levels, funding models, and the scope of nursing practice, may significantly impact nurse-provider communication, despite the countries’ comparable population demographics.

Clear and concise communication in the ED is essential for accurate diagnoses, prompt treatment, and appropriate disposition (Hettinger et al., 2020; Relias Learning, 2011). Communication breakdowns remain the leading cause of sentinel events in healthcare settings in the United States (The Joint Commission, 2023). For example, failure to report a change in a patient's condition, such as large fluctuations in their vital signs, may delay necessary interventions and result in deterioration and adverse outcomes, or may even be deadly (Amaniyan et al., 2020; The Joint Commission, 2023). Similarly, insufficient registered nurse-provider communication about the patient's diagnosis and care plan can result in inappropriate treatment or delays in care, which places patients at risk for harm (Amaniyan et al., 2020). Additionally, hierarchical dynamics may prevent nurses from voicing concerns about potentially unsafe provider orders or a lack of new orders (Eisenberg et al., 2007). This reluctance may be rooted in the perception that the provider always gets the “final say” and possesses authority over the diagnosis and establishment of the patient's care plan (Eisenberg et al., 2007).

This scoping review aimed to examine, systematically map, and identify gaps in existing literature concerning registered nurse-provider communication in the ED. A preliminary search using CINAHL, MEDLINE, and ProQuest Nursing & Allied Health revealed no systematic reviews or scoping reviews specific to this topic. Existing reviews have focused on nurse-provider communication more broadly (Tan et al., 2017), the impact of poor ED communication on patient safety (Amaniyan et al., 2020), and the effect of teamwork and communication training for ED staff on patient outcomes (Alsabri et al., 2022). However, given the unique communication dynamics and close collaboration between ED nurses and providers (Hettinger et al., 2020), this review sought to highlight key themes in existing literature and to define knowledge gaps that may require further investigation. With limited Canadian literature on ED nurse-provider communication, this review will also lay the groundwork for future research in Canada. Furthermore, synthesizing existing evidence on this topic will provide valuable insights that can inform policies targeting communication practices in the ED.

The primary research question guiding this scoping review was: What is the extent, nature, and range of literature currently available regarding registered nurse-provider communication in the ED? To answer this, three sub-questions were explored: (a) What content do registered nurses and providers feel is significant to include in nurse-provider communication to support the provision of high-quality care?, (b) What are the barriers and facilitators to registered nurse-provider communication in the ED?, and (c) To what extent do communication technologies (e.g., mobile devices, electronic health records) and organizational culture support or hinder registered nurse-provider communication in the ED?

## Methods and Procedures

A scoping review was chosen over other forms of systematic review because it allows for a topic to be broadly explored and for knowledge gaps to be identified after mapping existing literature (Munn et al., 2018). Scoping reviews accommodate diverse study designs (Munn et al., 2018), which is ideal given that nurse-provider communication in the ED remains under-researched and existing studies vary widely in methodology.

This review adhered to the JBI guidelines for scoping reviews (Peters et al., 2020) and was guided by Levac et al.'s (2010) six-stage framework: (i) identifying the research question, (ii) identifying relevant studies, (iii) study selection, (iv) data charting, (v) synthesizing and reporting results, and (vi) consultation with stakeholders. The optional consultation stage was not completed, as this review focused on analyzing and synthesizing existing literature. This approach promoted transparency and a systematic approach to mapping existing literature on nurse-provider communication in the ED.

### Eligibility Criteria

fIncluded sources highlighted direct communication between registered nurses and providers in the ED. *Communication* was defined as information exchanged between individuals using verbal, non-verbal, or electronic methods to achieve a shared understanding and facilitate apprcpriate decision-making (Coiera et al., 2002; The Joint Commission, 2023). This encompassed face-to-face communication, digital communication using electronic supports such as EHR-based messaging, and telephone communication. Nursing documentation and provider chart notes were excluded to emphasize real-time communication, which is predominant in the fast-paced ED, and to align with this review's focus on direct communication. Additionally, these were excluded as the literature shows providers do not typically review nursing documentation for decision-making (Amaniyan et al., 2020; Hettinger et al., 2020). The *emergency department* was broadly defined as a healthcare setting staffed 24/7 where nurses and providers care for high-acuity patients of any age with urgent or life-threatening conditions. Both hospital-based and free-standing EDs were included if they met these criteria.

This review evaluated sources focusing on direct communication between bedside RNs and providers, including MDs, PAs, and NPs in the ED. Practical nurses, such as registered practical nurses (RPNs) in Canada, were excluded from this review due to their limited presence in Canadian EDs. The ED is a rapidly changing, unpredictable environment with high turnover, and this less stable environment would require an RPN to consult and collaborate with an RN frequently, limiting autonomous practice (College of Nurses of Ontario [CNO], 2014). Within their scope, RPNs care for stable, lower-acuity patients autonomously (CNO, 2014), and many patients in the ED do not meet these criteria. Furthermore, this review excluded other members of the interdisciplinary healthcare team not mentioned above, and any nurses who were still in training or who were employed in leadership or management roles in which they did not provide direct patient care. This selective approach ensured that the included sources focused on direct patient care and frequent daily communication between nurses and providers, which aligned with the objectives of this review.

Only sources published in English were included to ensure consistent data extraction and analysis. Furthermore, given the limited availability of setting-specific literature found during an initial search related to this topic, this review did not impose any restrictions on the publication date of included sources. Commentaries and performance improvement projects were included alongside all relevant peer-reviewed literature. Peer-reviewed studies employing quantitative, qualitative, and mixed-methods designs, as well as any existing reviews were considered. Only sources specific to the ED setting were considered, and sources had to discuss nurse-provider communication. This comprehensive approach aimed to yield insights pertinent to clinical practice in the ED.

### Information Sources and Search Strategy

A two-part search strategy was utilized. First, a preliminary database search was conducted to find additional keywords and controlled terms to inform the final search strategy. Second, the full search strategy was run in MEDLINE ^®^ (Ovid), CINAHL (EBSCO), and ProQuest Nursing & Allied Health on August 19, 2024. These databases were chosen due to their comprehensive coverage of literature related to nurses and other health care professionals. The search strategy sought both key terms found in the titles, abstracts, and keyword fields and appropriate database-specific index terms (see Table 1 & Appendix).

### Selection of Sources of Evidence

After a search of the chosen databases, all identified sources were uploaded into Zotero v.6.0.37 (Corporation for Digital Scholarship and Roy Rosenzweig Centre for History and New Media, 2023). Using this software's duplicate removal feature, all duplicate sources were eliminated. Two reviewers independently screened the titles and abstracts of all imported sources for relevance, removing sources which did not appear to meet the eligibility criteria. The full texts of relevant sources identified through title-abstract screening were placed in a Zotero folder and reviewed by the authors to assess whether they met the inclusion/exclusion criteria. If a source met the exclusion criteria or failed to meet the inclusion criteria, it was then removed. Data extraction was then completed for all remaining sources.

### Data Extraction

This scoping review adhered to ethical standards outlined by JBI for conducting scoping reviews, ensuring transparency in source selection, data extraction and analysis accuracy (Peters et al., 2020). Because this review did not involve direct participation with human participants and only considered already published research and data from secondary sources, no institutional ethical approval was sought. Data were extracted by two independent reviewers from the selected papers using a blank version of a summary table developed by the primary author (see Supplemental Material). This table was designed following JBI recommendations for source information to be included in scoping reviews (Peters et al., 2020). Using a tabular format aligned with the scoping review's objectives, particularly the objective which was to evaluate the extent, nature, and range of existing literature concerning ED nurse-provider communication. Key details about each source, including information about their purpose, participants, methods, and key findings relevant to the review's objectives, were inputted into the full data extraction table (See supplemental material). Any discrepancies among reviewers were resolved through consensus and appropriate citations were used to respect intellectual property.

## Results

### Study Inclusion

**Figure 1.**
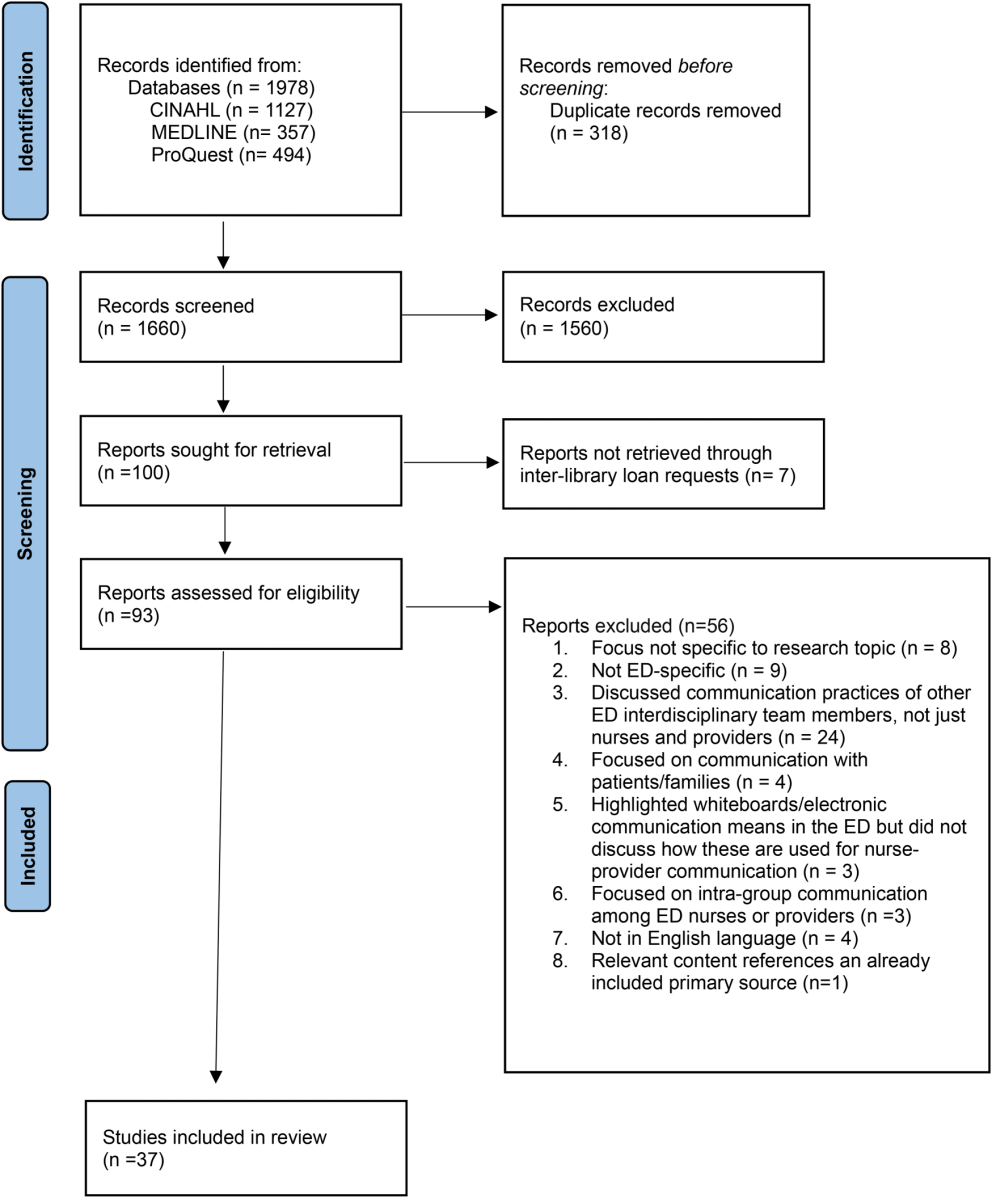
PRISMA- search results and study selection.

A total of 1978 sources were identified across three databases. After the removal of duplicates, 1680 titles and abstracts were screened, and 982 sources were excluded. Of the 100 remaining relevant sources, 93 underwent full-text review, though seven sources could not be retrieved. Following this, 56 studies were excluded for various reasons, such as addressing communication among other ED team members (*n* = 24), not addressing the research topic (*n* = 8), and not focusing on the ED setting (*n* = 9). Data was extracted from the remaining 37 sources for synthesis. Figure 1 illustrates the search results and inclusion/exclusion process in a PRISMA flow diagram.

### Characteristics of Included Sources

Of the 37 articles included in this review, the majority (*n* = 21) were from the United States, while the remaining sources spanned multiple international settings, including Australia, China, Saudi Arabia, and various European countries (See Table 2). Of note, only two studies were based in Canada, highlighting the limited literature addressing nurse-provider communication in Canadian EDs (Abourbih et al., 2015; Hacker Teper et al., 2022). The included sources employed different methodologies, including qualitative (*n* = 15), quantitative (*n* = 10), mixed methods (*n* = 4), commentaries (*n* = 6), and others (See Table 2).

### Results of Individual Sources of Evidence

Table 3 provides a summary of data extraction from the 37 included sources, and the full data extraction table is available as supplemental material.

**Table 1.** Example of database search strategy: MEDLINE via Ovid search results.

#	Query
1	((nurs* or “RN” or “RNs”) not (“nurse patient” or “nurse-patient” or “patient nurse” or “patient-nurse”)).ti,ab,kw.
2	nurses/
3	1 or 2
4	(physician* or “MD*” or doctor* or “physician assistant*” or “PA*” or “nurse practitioner*” or “NP*”).ti,ab,kw.
5	physicians/ or nurse practitioners/ or physician assistants/
6	4 or 5
7	(“emergency room” or “emergency department” or “emergency medicine” or “ER” or “ED”).ti,ab,kw.
8	exp Emergency Service, Hospital/
9	7 or 8
10	((“nurse physician” or “nurse-physician” or “physician patient” or “physician-patient” or “nurse-doctor” or “nurse doctor” or “doctor-nurse” or “doctor nurse” or “nurse provider” or “nurse-provider” or “provider nurse” or “provider nurse”) adj3 (communicat* or order* or report* or miscommunicat* or collaborat*)).ti,ab,kw.
11	communication/ or “cell phone use"/ or exp nonverbal communication/ or exp verbal behavior/
12	10 or 11
13	3 and 6 and 9 and 12

**Table 2.** Characteristics of included studies.

Study Characteristic	Number of Sources	References
Study Design		
Qualitative	15	Brixey et al., 2007; Brixey et al., 2008; Brixey et al., 2010; Coiera et al., 2002; Daouk-Öyry et al., 2017; Fairbanks et al., 2007; Hou et al., 2021; Gharaveis et al., 2018; Hettinger et al., 2020; Källberg et al., 2017; Leonardsen et al., 2024; Parizad et al., 2018; Pun et al., 2015; Relias Learning, 2011
Quantitative	10	Almulhim et al., 2020; Daheshi et al., 2023; Eppich, 2015; Gharaveis et al., 2020a; Hertzum, 2011; Luu et al., 2022; Schneider et al., 2019; Suryanto et al., 2016; Tindle et al., 2020; Weaver et al., 2017
Mixed Methods	4	Alameddine et al., 2015; Arianto & Jorgensen, 2010; Gharaveis et al., 2020b; Valentino et al., 2020
Commentary	6	Abourbih et al., 2015; Cunningham, 2021; O’Mara, 1999; Relias Learning, 2018; Relias Media, 2020; Williamson & Kives, 1991
Performance Improvement Project	1	Martin & Ciurzynski, 2015
Scoping Review	1	Hacker Teper et al., 2022
Country of Publication		
United States	21	Almulhim et al., 2020; Brixey et al., 2007; Brixey et al., 2008; Brixey et al., 2010; Daouk-Öyry et al., 2017; Eppich, 2015; Fairbanks et al., 2007; Gharaveis et al., 2018; Gharaveis et al., 2020a; Gharaveis et al., 2020b; Hettinger et al., 2020; Luu et al., 2022; Martin & Ciurzynski, 2015; O’Mara, 1999; Relias Learning, 2011; Relias Learning, 2018; Relias Media, 2020; Tindle et al., 2020; Valentino et al., 2020; Weaver et al., 2017; Williamson & Kives, 1991
Canada	2	Abourbih et al., 2015; Hacker Teper et al., 2022
Australia	2	Coiera et al., 2002; Suryanto et al., 2016
China	2	Hou et al., 2021; Pun et al., 2015
Denmark	2	Arianto & Jorgensen, 2010; Hertzum, 2011
South Africa	1	Cunningham, 2021
Sweden	1	Källberg et al., 2017
Saudi Arabia	1	Daheshi et al., 2023
Italy	1	
Norway	1	Leonardsen et al., 2024
Iran	1	Parizad et al., 2018
Germany	1	Schneider et al., 2019
Lebanon	1	Alamedinne et al., 2015

**Table 3.** Data extraction thematic analysis summary table.

Theme	Subtheme	Key Findings	Strategies/Solutions
Interruptions Hinder ED Nurse-Provider Communication	n/a	− Nearly a third of communication events are interruptions with an average range of 11.5–20.6 interruptions per hour (Brixey et al., 2010; Coiera et al., 2002) - ED nurses receive more patient comfort related interruptions than physicians (Schneider et al., 2019). Nurses’ interruptions related to coordination are closely related to patient's perceptions of ED wait times (Schneider et al., 2019). - ED physicians experience more multitasking alongside communication than nurses (14.6% vs 6.7%, respectively) (Coiera et al., 2002). - Interruptions were more frequent in the adult care area, and physicians faced more interruptions in comparison to bedside/charge nurses (Brixey et al. 2010) (6.9 per hour vs 0.5 and 3.9, respectively) (Fairbanks et al., 2007) - As care coordinators, ED RNs initiate more interruptions than physicians; however, they also receive more interruptions than they initiate (Brixey et al., 2007; Brixey et al., 2010). - Instead of interrupting, consider using asynchronous communication, when possible, for low-priority items (Hettinger et al., 2020) - ED RNs and MDs more often resume tasks than abandon them after being interrupted, usually completing only one interrupting activity before returning to their original task (Brixey et al., 2008; Brixey et al., 2010 - Interruptions contribute to information being lost and are more likely to result in communication breakdown during periods of high patient load (Källberg et al., 2017) -	- Weigh the importance of an interruption against its negative impact on smooth and efficient workflow (Brixey et al., 2008; Schneider et al., 2019) - A collaborative practice model allows for nurses to be well-informed of patients’ plans of care and results in fewer phone calls, pages, and interruptions to physicians (Williamson & Kives, 1991) - EPIC secure chat, an EHR-based messaging system shows promise in reducing nonurgent interruptions (Luu et al., 2022).
Barriers to ED Nurse-Provider Communication	Power Imbalance/ Culture of Hierarchy High Workload/Communication Load	- Hospital structures and organizational norms perpetuate professional separatism and a culture of hierarchy, contributing to a lack of self-perceived agency by nurses (Cunningham, 2021) - The power imbalance between ED nurses and physicians leads to mistrust, lower autonomy, and a lack of respect perceived by nurses, which increases moral distress. (Hou et al., 2021) - Hierarchy reduces nurses’ assertiveness in communication and causes them to use obliging conflict management and communication styles (Hou et al., 2021) - ED nurses over the age of 30, those with diplomas, over 10 years of experience and those in supervisory roles were more satisfied with ED nurse-provider communication (Daheshi et al., 2023) - Nurses and physicians of lower ED seniority disclose anxiety related to asking more senior clinicians for clarification/verification (Pun et al., 2015). More ED experience was linked to positive attitudes regarding nurse-provider collaboration (Suryanto et al., 2016) - Delegitimizing behaviours from providers, such as questioning nurses’ skills and expertise leads to a perceived loss of power by nurses (Parizad et al., 2018). - Hierarchal dynamics in the ED contribute to differences in cognitive load among nurses and providers, and maintaining traditional authority gradients leads to relational tensions. - ED nurses and providers face a high cognitive load and communicate frequently in a fast-paced, dynamic setting (Cunningham, 2021). This can lead to fatigue and stress which encourage verbal altercations and conflict (Parizad et al., 2018) - RN-provider communication is highly frequent in the ED, but it is often rapid due to time constraints and the sheer volume of information (Pun et al., 2015) - ED RNs and physicians view high workloads as the main cause of communication failures, including a lack of shared information and flaws in information delivery/receipt. (Källberg et al. (2017) - Inadequate ED staffing and high workloads can make it difficult for nurses to contact providers when care needs to be escalated, and for providers to respond in a timely manner (Hacker Teper et al., 2022; Parizad et al., 2017)	- Recognize and appreciate the value of ED nurses’ contributions and foster open nurse-provider communication and collegial relationships through strong management support (Cunningham, 2021; Daheshi et al., 2023; O’Mara, 1999; Parizad et al., 2017) - Physicians should informally introduce themselves to help eliminate hierarchy (Abourbih et al., 2015) - Create an environment of shared learning and mutual respect (Abourbih et al., 2015) - Create a climate of psychological safety, in which all team members feels safe and supported to voice concerns and provide input (Eppich, 2015) - Keep communication succinct, clear, and direct (Abourbih et al., 2015; Daouk-Öyry et al. (2017) - Use integrated communication channels to increase communication efficiency (Cunningham, 2021) - Structured communication tools, such as SBAR, can help ED nurses present the urgency of a patient situation when contacting the ED provider (Hacker Teper et al., 2022) - Don’t assume another team member is already aware of a piece of information (Daouk-Öyry et al., 2017; Hettinger et al. 2020)
Facilitators to ED Nurse-Provider Communication	Shared/High Visibility Workspaces Electronic Supports Collaborative, Respectful Interactions	- Most ED communication is face-to-face, and informal in nature (Brixey et al., 2008; Coiera et al., 2002; Gharaveis et al., 2018), and most communication events occur in the nurse-physician workstation (Fairbanks et al., 2007) - There is a significant positive correlation between increased visibility of central areas in the ED, such as workstations, and successful ED nurse-provider communication and optimal outcomes (Gharaveis et al., 2020a). - Visibility is significant as it improves teamwork and communication, and it reduces distractions during communication, thus improving concentration (Gharaveis et al., 2018; Gharaveis et al., 2020b) - Shared nurse-provider workstations improved timeliness and clarity of communication, while also improving situation monitoring and allowing team members to better anticipate needs (Weaver et al., 2017). - If physicians and nurses do not share workspaces, this limits physical proximity, increases wasted movement (Almulhim et al., 2020) and reduces situational awareness (Hettinger et al., 2020) - Centrally placed electronic whiteboards can be useful for information sharing between ED RNs and providers, particularly in sections of the ED where patient stay is longer (Hertzum, 2011) - EHR features such as EPIC secure chat allow for communication of non-urgent issues while maintaining workflow and reducing interruptions/ED physician burnout (Luu et al. 2022) - Best practice advisories and system triggers can prompt ED RNs to consider the need to re-evaluate vital signs before discharge/and or to notify the provider, facilitating appropriate clinical decision making (Relias Learning, 2011; Valentino et al., 2020) - ED nurses work closely with providers and need to feel comfortable collaborating with them (Daheshi et al., 2023) - Appreciating and acknowledging your colleagues helps to create a collegial environment of shared learning (Abourbih et al., 2015; O’Mara, 1999) - Strive to communicate effectively to establish a shared vision for the patient's plan of care (Cunningham, 2021) - Directly address concerns with ED providers and use open communication to resolve disagreements (Relias Media, 2020) - Use a kind, respectful tone; use professional language; maintain eye contact, and actively listen (Daouk-Öyry et al., 2017) - Use team huddles and joint training to allow RNs and providers to become familiar with each other's contributions and facilitate relationship-building (Daheshi et al., 2023; Hettinger et al., 2020)	- When possible, shared workstations or central areas with high visibility between nurses and providers should be incorporated into ED layouts to promote effective communication (Gharaveis et al., 2020a; Tindle et al., 2020; Weaver et al., 2017). This can help to foster collaboration in the era of digitalization, which creates physical distance among ED providers (Leonardsen et al., 2024) For tasks that need to be done immediately/critical patient information, use verbal communication to supplement EHR charting (Hettinger et al., 2020) Use spoken/written confirmation of receipt/understanding for all interdisciplinary communication (Pun et al., 2015). Otherwise, nurses may not receive messages or respond in a timely way with their actions (Leonardsen et al., 2024) - Joint nurse-provider patient evaluations followed by a huddle to discuss the treatment plan and roles can be helpful (Martin & Ciurzynski (2015) - Provide opportunities for ED RNs and providers to improve their communication skills, such as through interprofessional training (Alameddine et al., 2015; Coiera et al., 2002; O’Mara, 1999; Relias Learning, 2011; Suryanto et al., 2016) - A joint-practice committee or a nurse-provider forum can allow for interprofessional discussion and planning (Williamson & Kives, 1991)
ED Nurse-Provide Communication: Valued Content	What ED Providers Want to Know from ED Nurses What ED Nurses Want to Know from ED Providers Information of Mutual Interest	- Changes in patient condition; abnormal assessment findings/ vital signs (Hettinger et al., 2020). This is particularly important if a patient is to be discharged (Relias Learning, 2011). - When proactive testing/therapeutic interventions are needed before clinical evaluation (Hettinger et al., 2020) - When order clarification is required (Abourbih et al., 2015) - Assessment findings/the plan of care (Arianto & Jorgensen, 2010; Hettinger et al., 2020) - Any changes to the plan of care so the patient & family can be updated (Pun et al., 2015) - The rationale behind orders (Abourbih et al., 2015) - The patient's disposition (discharge vs observe vs admit) (Abourbih et al., 2015; Arianto & Jorgensen, 2010), - Outstanding tasks/remaining steps in a patient's care (Hettinger et al., 2020) - Experience level/specialized knowledge you can contribute to shared learning (Abourbih et al., 2015)	- ED nurses should always document in a timely manner, but they must also recognize the provider may not see their note and use other means of communication to relay important information that is urgent (Relias Learning, 2018)

### Synthesis of Results

The 37 sources were analyzed for similarities and differences. The authors reviewed key data extracted from each source several times to become familiar with it and immerse themselves in it, highlighting repeated ideas, and grouping them accordingly. For the text from each source, thematic analysis was used to code themes related to the research objectives, and these were then revised and named (Braun & Clarke, 2006). Four major themes emerged relating ED registered nurse-provider communication: (i) Interruptions hinder communication (ii) Power imbalances and high workload/communication load act as barriers (iii), Shared, high visibility workspaces, electronic supports, and collaborative, respectful interactions are facilitators (iv) Valued content includes timely updates and collaborative planning. Concept maps were developed for each theme to provide a visual representation (See Supplemental Material).

#### Interruptions Hinder ED Registered Nurse-Provider Communication

Almost one-third of nurse-provider communication events in the ED are interruptions (Brixey et al., 2010; Coiera et al., 2002). Interruptions can contribute to information being lost and are more likely to result in communication breakdown during periods of high patient load in the ED (Källberg et al., 2017). Most interruptions are initiated by ED nurses, which may be due to the nature of their role as care coordinators; however, despite this, ED nurses still receive more interruptions than they initiate (Brixey et al., 2007). However, ED physicians experience more interruptions than ED nurses, resulting in a higher percentage of communication events involving multitasking among physicians (14.6%) compared to nurses (6.7%) (Brixey et al., 2007; Coiera et al., 2002; Fairbanks et al., 2007). After being interrupted, ED RNs and providers typically resume tasks rather than abandoning them, usually completing only one interrupting activity before resuming their original task (Brixey et al., 2008; Brixey et al., 2010). Additionally, there are role-related differences in the content of interruptions; ED nurses more often receive interruptions related to patient comfort than physicians (Schneider et al., 2019). Aspects of the ED design, including whether supplies and equipment are readily available in the immediate workspace for nurses and providers also contribute to interruptions, as stepping away to obtain a supply may result in an interruption from a member of the care team in the hallway (Brixey et al., 2008).

#### Barriers to ED Registered Nurse-Provider Communication

##### Power imbalance/culture of hierarchy

The power differential between ED nurses and providers, reinforced by hospital structures and hierarchical organizational norms, limits nurses’ assertiveness and can negatively impact communication (Cunningham, 2021; Hou et al., 2021). Hierarchical dynamics may lead nurses to adopt obliging conflict management styles, which can foster mistrust and a lack of respect, ultimately increasing moral distress (Hou et al., 2021). Additionally, maintaining traditional authority gradients leads to relational tensions and delegitimizing behaviours from ED providers, such as questioning nurses’ skills and expertise, leading to nurses’ perceived loss of power (Parizad et al., 2018). Previous encounters with disruptive or unprofessional behaviour reduce the likelihood of nurses feeling comfortable asking questions or sharing concerns in the ED, which can jeopardize patient safety (Eppich, 2015). To improve communication, ED providers must recognize and appreciate nurses’ contributions to the interprofessional team and patient care (Cunningham, 2021; O’Mara, 1999). When nurses feel their opinions are valued, they are more likely to provide input and are less likely to be frustrated in the workplace (O’Mara, 1999).

Interestingly, ED nurses are not the only ones negatively impacted by the culture of hierarchy; Pun et al. (2015) found that both nurses and providers in the ED experienced anxiety related to asking more senior clinicians for clarification/verification. Supporting the idea that participants’ seniority level impacts nurse-provider communication, Daheshi et al. (2023) found that nurses with at least 10 years of experience, nurses over the age of 30, nurses who hold a diploma, and nurses working in supervisory roles are more satisfied with ED nurse-provider communication. Likewise, Suryanto et al. (2016) found that nurses and physicians with more ED experience had more positive attitudes regarding nurse-provider collaboration, potentially because they were more comfortable in the ED environment.

##### High Workload/ Communication Load

ED nurses and providers face a high cognitive load and communicate frequently in a high-stress setting (Cunningham, 2021). Although RN-provider communication is frequent in the ED, with an average of 36.5 communication events per hour (Coiera et al., 2002), it is often rapid due to time constraints and the sheer volume of information (Pun et al., 2015). ED RNs and physicians view high workloads as the main cause of communication failures, including a lack of shared information and flaws in information delivery/receipt. (Källberg et al., 2017). Inadequate ED staffing and high workloads can make it difficult for nurses to contact providers when care needs to be escalated, and for providers to respond promptly (Hacker Teper et al., 2022). This can lead to fatigue and stress which encourage verbal altercations and conflicts (Parizad et al., 2018). Furthermore, despite both interprofessional groups facing large workloads, hierarchical dynamics in the ED have been linked to differences between nurses’ and providers’ cognitive loads.

#### Facilitators to ED Registered Nurse-Provider Communication

##### Shared/high visibility workspaces

Shared workspaces are important in the ED as the majority of ED nurse-provider communication is face-to-face and informal (Brixey et al., 2008; Coiera et al., 2002; Gharaveis et al., 2018), with most communication events occurring in the nurse-physician workstation (Fairbanks et al., 2007). Without shared workspaces, physical proximity is limited, which increases wasted movement (Almulhim et al., 2020), reduces situational awareness (Hettinger et al., 2020), and leads to most interactions occurring in the patient's room (Tindle et al., 2020). A significant positive correlation exists between increased visibility of central areas in the ED, such as workstations, and successful ED nurse-provider communication and optimal outcomes (Gharaveis et al., 2020a). Visibility is important as it improves teamwork and communication and it reduces distractions during communication, thus improving concentration (Ghraraveis et al., 2018; Gharaveis et al., 2020b). Shared nurse-provider workstations improve the timeliness and clarity of communication, while also improving situation monitoring and allowing team members to better anticipate needs (Weaver et al., 2017). They can help to foster interprofessional collaboration, particularly in modern EDs, where communication mainly occurs through digital messaging which creates physical distance among providers (Leonardsen et al., 2024).

##### Electronic supports

Emerging technologies can help to facilitate effective communication among ED nurses and providers. During times of high patient census in the ED, when nurses and providers are busy and physically distant from each other, electronic tools can facilitate faster communication than face-to-face methods (Hacker-Teper et al., 2022). This is particularly crucial when a patient's condition is deteriorating and timely escalation of care is needed (Hacker Teper et al., 2022). For example, centrally placed electronic whiteboards can be useful for information sharing between ED RNs and providers, particularly in sections of the ED where patient stay is longer and there is more evolving information (Hertzum, 2011). Another way in which technology can benefit ED nurse-provider communication is through the implementation of best practice advisories (BPAs) and safety triggers into the EHR. For instance, a BPA or a system trigger can facilitate appropriate clinical decision-making and communication by prompting ED RNs to consider the need to re-evaluate vital signs and/or notify the provider before discharge (Relias Learning, 2011; Valentino et al., 2020). Similarly, EPIC secure chat is an EHR-based feature that allows for communication of non-urgent issues while maintaining workflow and reducing interruptions and physician burnout (Luu et al., 2022).

Despite how useful electronic supports are for nurse-provider communication, spoken/written confirmation of receipt/understanding should be used for all interdisciplinary ED communication that occurs online to prevent errors (Pun et al., 2015). Otherwise, the content of the digital communication may not be received and responded to promptly (Leonardsen et al., 2024). Additionally, for tasks that need to be done immediately by either the nurse or the provider, and for critical patient information, verbal communication should always be used in addition to online means to facilitate timely intervention (Hettinger et al., 2020).

##### Collaborative, respectful interactions

The goal of ED nurse-provider communication should be to create a shared vision for the patient's plan of care (Cunningham, 2021) while building mutual respect (Abourbih et al., 2015). Any concerns should be directly addressed by nurses and providers and open communication should be used to foster effective resolution of any nurse-provider disagreements (Relias Media, 2020). To encourage positive interactions, both parties should appreciate and acknowledge their colleagues, creating a collegial environment of shared learning (Abourbih et al., 2015; O’Mara, 1999). In daily practice, joint evaluation of the patient by the ED nurse and provider followed by a huddle to discuss the treatment plan and roles can help to facilitate collaborative dynamics (Martin & Ciurzynski, 2015).

#### ED Registered Nurse-Provider Communication: Valued Content

##### What ED nurses want to know from ED providers

Intending to provide optimal patient care, ED nurses prioritize understanding certain aspects of communication from ED providers. They want to be notified by providers of key assessment findings and the patient's plan of care (Hettinger et al., 2020). An initial dialogue between the provider and nurse about the treatment a patient will require while in the ED allows for a shared plan of care to be developed, resulting in more efficient ED patient care delivery (Arianto & Jorgensen, 2010). As well, ED nurses feel that understanding the rationale behind the provider's orders is crucial (Abourbih et al., 2015), as this can help them to prioritize interventions appropriately. Any changes to the plan of care should be promptly conveyed by the provider to allow for re-prioritization of tasks and timely updates to the patient and family (Pun et al., 2015). Lastly, ED nurses value knowing the patient's disposition as soon as possible, including whether they will be discharged, observed in the ED, or admitted, as this can help in planning care (Abourbih et al., 2015)

##### What ED providers want to know from ED nurses

ED providers prioritize knowing several key aspects of communication from ED nurses to facilitate effective patient care. Providers want ED nurses to update them promptly regarding any changes in a patient's condition, including abnormal assessment findings and abnormal vital signs (Hettinger et al., 2020). System triggers can alert ED nurses and physicians of abnormal vital signs and changes in a patient status, which facilitates a prompt response to unstable patients (Relias Learning, 2011). For example, after noting critical abnormalities upon the patient's arrival to the ED, the triage RN can activate the team to ensure that ED physicians and other nurses to immediately respond to the patient's needs (Relias Learning, 2011). However, when relying on documentation in the electronic health record to communicate with the ED provider, nurses must recognize that, although their entry may be documented promptly, the provider most often does not get notified an entry has been added and may not read the entry for some time (Relias Learning, 2018). Face-to-face conversation or a telephone call should be considered for pressing information (Relias Learning, 2018). ED providers also want to be notified when nurses feel that proactive testing or therapeutic interventions are required before they can come to evaluate the patient (Hettinger et al., 2020). Additionally, providers want to be informed when order clarification is required (Abourbih et al., 2015).

##### Information of mutual interest

Both ED nurses and providers value collaborative communication and aim to discuss outstanding tasks and remaining steps in a patient's care to improve workflow (Hettinger et al., 2020). As well, both parties sharing specialized knowledge and past experiences can contribute to a shared learning environment (Abourbih et al., 2015).

## Discussion

Effective ED nurse-provider communication is crucial to high-quality patient care but remains challenging due to the high-stress, dynamic nature of the ED. This scoping review mapped existing literature on registered nurse-provider communication in the ED, identifying key themes and knowledge gaps.

### Addressing Barriers to ED Nurse-Provider Communication

Interruptions were a significant communication challenge for ED nurses and providers, particularly during high patient census (Källberg et al., 2017). Asynchronous communication tools, such as EHR-based messaging, may reduce interruptions due to non-urgent matters (Coiera et al., 2002; Luu et al., 2022), but can also increase accessibility demands on nurses and providers (Brixey et al., 2008), Using a collaborative practice model can reduce interruptions as it ensures nurses are well-informed of patients’ plans of care, resulting in fewer phone calls, pages, and interruptions to physicians (Williamson & Kives, 1991). Before initiating an interruption, both ED nurses and providers should weigh the importance of the interruption against any negative impact it may have on the efficiency of the ED workflow (Brixey et al., 2008).

Given the high communication and workloads ED nurses and providers are challenged by, integrated communication tools, such as SBAR, should be the standard within organizations to help streamline communication and to ensure that important information is conveyed clearly and efficiently (Cunningham, 2021; Hacker Teper et al., 2022). These tools can help ED nurses to present the urgency of a patient situation and escalate care, when necessary, in collaboration with the ED provider (Hacker Teper et al., 2022)

Another significant barrier to effective ED nurse-provider communication identified in this review was the power differential and hierarchical culture between ED nurses and providers. Research suggests that this power imbalance impairs effective communication, especially when nurses feel they lack agency (Cunningham, 2021). In such situations, nurses are less likely to voice concerns or question provider orders, which can pose significant risks to patient care and safety (Cunningham, 2021).

To address power imbalances and reduce hierarchy in the ED, small behavioural adjustments, such as physicians introducing themselves to nurses by first name, can go a long way (Abourbih et al., 2015). On an organizational level, creating a culture of respect and collaboration, where both nurses and providers recognize each other's contributions to patient care and expertise should be a priority (Abourbih et al., 2015; Hou et al., 2021). Fostering opportunities for interprofessional communication, such as joint case debriefings and shared huddles, can help achieve this by promoting shared decision-making and mutual respect among both groups (Daheshi et al., 2023; Hettinger et al., 2020; Martin & Ciurzynski, 2015).

Additionally, establishing joint-practice committees or nurse-provider forums can allow for formal interprofessional discussion and planning, further reducing hierarchical dynamics (Williamson & Kives, 1991). These provide opportunities for nurses and providers to become more familiar with one another's roles, levels of experience, and unique contributions to the team, thus building collegial relationships and establishing a shared sense of responsibility for patient care (Daheshi et al., 2023; Hettinger et al., 2020).

Mitigating nurse-provider power imbalances in the ED also requires strong organizational support. ED leadership should role model respect for all ED team members, regardless of their role (Daheshi et al., 2023) and enforce ‘zero-tolerance’ policies for hierarchical behaviours - such as providers assuming superiority over nurses- to ensure a culture of mutual respect and equity.

### Facilitators of Effective Communication

Facilitators of effective ED nurse-provider communication identified in this review include shared workstations, electronic supports and collaborative, respectful interactions. Shared workstations are critical as they promote face-to-face interactions and increase situational awareness (Fairbanks et al., 2007; Gharaveis et al., 2020a). Thus, shared workstations or central areas with high visibility between nurses and providers should be incorporated into ED layouts to promote effective communication (Gharaveis et al., 2020a; Tindle et al., 2020; Weaver et al., 2017). Electronic supports, such as centrally placed electronic whiteboards and EHR-based secure chat features, should be implemented in EDs as they have been shown to facilitate the sharing of information and care coordination, thus reducing communication gaps, and fostering teamwork (Gharaveis et al., 2020a; Hertzum, 2011).

Consistent with the review objective, communication content valued by both ED nurses and providers was identified. ED nurses value providers communicating notable assessment abnormalities after their clinical evaluation (Hettinger et al., 2020). They also need to understand the rationale behind provider orders, seek clarification as needed, and value communication regarding the patient's plan for disposition (Abourbih et al., 2015; Hettinger et al., 2020). Conversely, ED providers prioritize prompt updates on changes in patient condition, including abnormal assessment findings and vital signs, proactive testing or therapeutic needs, and order clarification (Abourbih et al., 2015; Hettinger et al., 2020). Clear and timely communication of these details is key to providing safe and efficient care.

### Implications for Policy and Practice

Integration of Technology: Integrating electronic supports to optimize communication between nurses and providers in the ED can reduce interruptions, enhance workflow, and improve information transfer (Hacker Teper et al., 2022; Hettinger et al., 2020; Luu et al., 2022; Valentino et al., 2020). Despite the benefits of electronic support, such as EHRs and cell phones for nurses and providers, these tools are not consistently utilized. For instance, EHR implementation disparities exist among the Canadian provinces, as over 60% of Alberta and Ontario EDs use EHRs, while EDs in Quebec and Newfoundland have adoption rates below 40%, (Canada Health Infoway, 2016). These technologies should be more consistently implemented to improve communication and patient safety. Furthermore, when using electronic supports to communicate, it is key that both ED nurses and providers never assume that the other party is already aware of a piece of patient information, as this can lead to the omission of crucial details (Hettinger et al. 2020; Pun et al., 2015). Providers should follow up with nurses to ensure understanding and adherence to communication (Daouk-Öyry et al., 2017)

Education and Training: Implementing strategies aimed at reducing hierarchical culture and providing interprofessional communication training can hone ED nurses’ and providers’ communication skills, fostering collaborative relationships (Alameddine et al., 2015; Coiera et al., 2002). Crew Resource Management (CRM), an intervention originating from the aviation field, has gained popularity in healthcare as it emphasizes flattening hierarchies to foster open communication among team members of various ranks to reduce errors related to communication failure (Buljac-Samardzic et al., 2021). Such interventions should be incorporated into organizational training and professional development initiatives to demonstrate ongoing dedication to reducing power imbalances and improving nurse-provider relationships.

Organizational Support: Hospital administrations should acknowledge the importance of shared nurse-provider workspaces and consider redesigning EDs to incorporate them (Gharaveis et al., 2020a; Weaver et al., 2017). Organizational policies that promote open, respectful nurse-provider communication should be implemented to demonstrate institutional commitment to this significant aspect of patient care. Additionally, strong support from management is key to establishing an environment that values nurses’ contributions, fosters psychological safety, and encourages nurses to provide input and voice concerns without hesitation (Daheshi et al., 2023). Likewise, organizational leaders should address high workloads, a substantial barrier to effective ED nurse-provider communication, by implementing responsive staffing models that recognize peak ED demand times to help reduce communication failure and mitigate gate burnout (Hacker Teper et al., 2022).

### Limitations

This review had several limitations which must be considered when evaluating its findings. Most of the included sources investigated nurse-provider communication in American healthcare settings, with only two Canadian sources (Abourbih et al., 2015; Hacker Teper et al., 2022) and a limited number of international sources. Thus, generalizing findings to other geographical locations, such as Canada, where healthcare systems are structured differently, and where healthcare policy, culture, and patient demographics differ, may not be possible. The exclusion of non-English studies further limits the generalizability of this review's findings on a global scale.

Additionally, because this review did not include a grey literature search, guidelines, reports, or unpublished sources that align with the review's inclusion criteria may have been excluded by omission. Furthermore, because only three databases were searched, this potentially excluded relevant sources indexed in other databases. Therefore, the comprehensiveness of this review's findings cannot be confirmed.

## Conclusion

This scoping review grants insight into the significance of effective nurse-provider communication in the fast-paced ED, emphasizing facilitators, such as shared workspaces and electronic supports, and identifying barriers, including communication interruptions and hierarchical dynamics. To address these challenges, it is recommended EDs integrate electronic communication tools, implement structured communication protocols, such as SBAR, foster mutual respect through shared huddles and joint debriefings, and adopt responsive staffing models. Furthermore, organizations should prioritize interprofessional communication training to foster collaboration and work towards infrastructure that supports shared workspaces for ED nurses and providers.

Given the predominance of American studies and the scarcity of Canadian research on this topic, localized research and studies focusing on regional and provincial differences are needed to better understand the unique barriers and facilitators to ED nurse-provider communication in Canadian EDs. Such research would inform the development of contextually relevant interventions and allow for meaningful comparison with international findings. Future studies should also assess the long-term sustainability of communication interventions, such as technology integration, to evaluate how these influence workflow, interprofessional communication, and care quality in the ED.

## Supplemental Material

sj-docx-1-cjn-10.1177_08445621251320710 - Supplemental material for Barriers and Facilitators to Nurse-Provider Communication in the Emergency Department: A Scoping Review Supplemental material, sj-docx-1-cjn-10.1177_08445621251320710 for Barriers and Facilitators to Nurse-Provider Communication in the Emergency Department: A Scoping Review by Sylwia Borawski, Jody Ralph and Adam Mulcaster in Canadian Journal of Nursing Research

## Appendix

### Search results

**Table 1A.** MEDLINE via Ovid search results.

#	Query	Results from 19 Aug 2024
1	((nurs* or “RN” or “RNs”) not (“nurse patient” or “nurse-patient” or “patient nurse” or “patient-nurse”)).ti,ab,kw.	566,830
2	nurses/	47,640
3	1 or 2	578,895
4	(physician* or “MD*” or doctor* or “physician assistant*” or “PA*” or “nurse practitioner*” or “NP*”).ti,ab,kw.	20,312,447
5	physicians/ or nurse practitioners/ or physician assistants/	128,696
6	4 or 5	20,346,807
7	(“emergency room” or “emergency department” or “emergency medicine” or “ER” or “ED”).ti,ab,kw.	317,297
8	exp Emergency Service, Hospital/	104, 132
9	7 or 8	356,878
10	((“nurse physician” or “nurse-physician” or “physician patient” or “physician-patient” or “nurse-doctor” or “nurse doctor” or “doctor-nurse” or “doctor nurse” or “nurse provider” or “nurse-provider” or “provider nurse” or “provider nurse”) adj3 (communicat* or order* or report* or miscommunicat* or collaborat*)).ti,ab,kw.	1,718
11	communication/ or “cell phone use"/ or exp nonverbal communication/ or exp verbal behavior/	182,453
12	10 or 11	183,500
13	3 and 6 and 9 and 12	357

*Notes:* Platform: Ovid; Database: MEDLINE ^®^ and Epub Ahead of Print, In-Process, In-Data Review & Other Nonindexed Citations, Daily and Versions <1946 to August 15, 2024>; Search Date: August 19, 2024.

**Table 2A.** CINAHL via EBSCOhost search results.

#	Query	Results
S13	S3 AND S6 AND S9 AND S12	1,127
S12	S10 OR S11	368,389
S11	(MH “Communication+”) OR (MH “Nonverbal Communication+”) OR (MH “Cell Communication”) OR (MH “Reports+”) OR (MH “Medical Orders”) OR (MH “Nursing Orders”)	367,840
S10	(((((TI((“nurse physician” or “nurse- physician” or “physician patient” or “physician-patient” or “nurse-doctor” or “nurse doctor” or “doctor-nurse” or “doctor nurse” or “nurse provider” or “nurse-provider” or “provider nurse” or “provider-nurse”) N3 (communicat* or order* or report* or miscommunicat* or collaborat*).))))) OR (((((AB((“nurse physician” or “nurse- physician” or “physician patient” or “physician-patient” or “nurse-doctor” or “nurse doctor” or “doctor-nurse” or “doctor nurse” or “nurse provider” or “nurse-provider” or “provider nurse” or “provider-nurse”) N3 (communicat* or order* or report* or miscommunicat* or collaborat*).)))))	1,021
S9	S7 or S8	160, 027
S8	(MH “Emergency Medicine”) OR (MH “Emergency Service”) OR (MH “Emergency Care”)	96,663
S7	(((TI (“emergency room” or “emergency department” or “emergency medicine” or “ER” or “ED”)))) OR (((AB (“emergency room” or “emergency department” or “emergency medicine” or “ER” or “ED”))))	112,223
S6	S4 or S5	4,136,114
S5	(MH “Physicians, Emergency”) OR (MM “Physicians”) OR (MM “Nurse Practitioners”) OR (MM “Physician Assistants”)	55,911
S4	(((TI (physician* or “MD*” or doctor* or “physician assistant*” or “PA*” or “nurse practitioner*” or “NP*”)))) OR (((AB(physician* or “MD*” or doctor* or “physician assistant*” or “PA*” or “nurse practitioner*” or “NP*”))))	4,118,081
S3	S1 or S2	635,322
S2	(MH “nurses”)	73,884
S1	((((TI((nurs* or “RN” or “RNs”) not (“nurse patient” or “nurse-patient” or “patient nurse” or “patient-nurse”)))))) OR ((((AB((nurs* or “RN” or “RNs”) not (“nurse patient” or “nurse-patient” or “patient nurse” or “patient-nurse”))))))	602,456

*Notes:* Platform: EBSCO; Database: CINAHL; Search Date: August 19, 2024.

**Table 3A.** Proquest search results.

#et#	Query	Results
S1	TIAB((nurs* or “RN” or “RNs”) not (“nurse patient” or “nurse-patient” or “patient nurse” or “patient-nurse”))	315393
S2	MAINSUBJECT.EXACT(“Nurses”)	139527
S3	([S1] OR [S2])	348315
S4	TIAB(physician* or “MD*” or doctor* or “physician assistant*” or “PA*” or “nurse practitioner*” or “NP*”)	1,849,721
S5	MAINSUBJECT.EXACT(“Physicians”) OR MAINSUBJECT.EXACT(“Nurse practitioners”) OR MAINSUBJECT.EXACT(“Physician assistants”)	120,656
S6	([S4] OR [S5])	1,885,181
S7	TIAB(“emergency room” or “emergency department” or “emergency medicine” or “ER” or “ED”)	84,772
S8	MAINSUBJECT.EXACT(“Emergency medical care”)	71,832
S9	([S7] OR [S8])	126,223
S10	TIAB((“nurse physician” or “nurse-physician” or “physician patient” or “physician-patient” or “nurse-doctor” or “nurse doctor” or “doctor-nurse” or “doctor nurse” or “nurse provider” or “nurse-provider” or “provider nurse” or “provider nurse”) N/3 (communicat* or order* or report* or miscommunicat* or collaborat*))	579
S11	MAINSUBJECT.EXACT(“Communication”) OR MAINSUBJECT.EXACT(“Verbal communication”)	50,537
S12	([S10] OR [S11])	50,885
S13	([S3] AND [S6] AND [S9] AND [S12])	494

*Notes:* Platform: ProQuest; Database: ProQuest Nursing and Allied Health Source; Search Date: August 19, 2024.
