# Supplementary material for: Barriers and Facilitators to Nurse-Provider Communication in the Emergency Department: A Scoping Review
Source: Can J Nurs Res. 2025 Mar 17;57(2):267–83. doi: 10.1177/08445621251320710 (PMC12086286; doi:10.1177/08445621251320710)
Supplement: sj-docx-1-cjn-10.1177_08445621251320710 - Supplemental material for Barriers and Facilitators to Nurse-Provider Communication in the Emergency Department: A Scoping Review [file sj-docx-1-cjn-10.1177_08445621251320710.docx]

**Supplemental Material**

**Full Data Extraction Table**

*Literature Regarding Registered Nurse-Provider Communication in the Emergency Department (ED)*

| Author(s), Year, Country | Purpose | | Literature Source Type/Design | | Setting and Sample | | Methods | | Key Findings | |  |
| --- | --- | --- | --- | --- | --- | --- | --- | --- | --- | --- | --- |
| Abourbih et al. (2015), Canada | To provide strategies for improved communication among ED nurses and providers to facilitate optimal patient outcomes. | | Commentary | | Not applicable to this type of literature source. | | Not applicable to this type of literature source. | | - Nurses and providers working in the ED often have similar personalities and are drawn to its’ fast-paced environment. - Building effective communication and appreciating and acknowledging your colleagues can help to optimize success and prevent burnout.   Suggestions for ED Providers:   - Introduce yourself to the nurses in an informal way, preferably by first name, to help eliminate any hierarchy/superiority and create a collegial environment. - Consult the nurse before your assessment of the patient. - Share your knowledge when appropriate. - Explain the rationale behind your orders. - Include nurses in reassessments and patient hand-over between providers.   Suggestions for ED Nurses:   - Introduce yourself to the provider. - Embrace new learners, including medical residents or less experienced providers. - Be direct and clear in your communication, asking for clarification when needed. - If communication breakdown occurs, search for solutions to the barriers to effective communication. - Create an environment of shared learning with providers | |  |
| Alameddine et al. (2015), Lebanon | | To explore the advantages and disadvantages of using peer assessments by nurses to evaluate the non-clinical performance of ED physicians; To identify what remedial interventions would foster sustainable improvements and lasting success. | | Sequential mixed methods design | | - The ED in one of Lebanon’s largest academic centers. - Three evaluations with 27, 33, and 28 nurses being asked to evaluate 20, 21, and 23 physicians, respectively. - Most physicians were male (81.5%) | | - Secondary analysis using a de-identified database that was created from a cross-sectional survey of ED registered nurses. - The survey asked for their peer evaluation of ED physicians’ professionalism, communication, and leadership skills. - Three evaluations were completed over an 18-month period. - Physicians underwent communication training after the first evaluation and received in-depth feedback after all three evaluations. - Paired t-tests were used to compare mean scores among the three evaluation cycles. - Thematic analysis was used for qualitative comments. | | - Evaluations of physicians’ non-clinical performance by nurses can improve the communication, leadership skills, and professionalism of physicians. - Evaluation scores improved most between the first and second evaluations; notably, this was after communication training was provided. - Improvements were not sustained to the third evaluation, suggesting the need for a booster/refresher course. - The effects of such interventions may be diluted by the ED’s intense environment and heavy workloads, and the acquired desirable behaviour being forgotten. | |
| Almulhim et al. (2020), United States | | To discuss how three different ED care team assignments (static geographic, dynamic physician-nurse and geographic nursing-physician) impact efficiency, communication, and patient outcomes. | | Comparative analysis | | Not applicable to this study design | | - The authors developed eight performance criteria to compare the three ED staff teaming models: ease of patient assignment, speed of patient assignment, physician-nurse communication, physician-nurse synchrony, incentives aligned for patient flow, limits wasted movement, load balancing of teams, and flexibility. | | - If weighing all factors equally, with each category having a maximum score of four points, the static geographic model scores 23 points, outranking the dynamic physician nurse (21 points) and geographic nursing-physician (15 points) models. - In the static geographic staff teaming model, ED nurses and physicians often sit at a shared workstation, and this proximity results in less wasted movement and improved nurse-physician communication. However, this model offered less flexibility in comparison to the other two models. | |
| Arianto & Jorgensen (2010), Denmark | | To explore how optimizing communication between nurses and doctors can reduce ED wait times. | | Exploratory sequential mixed-methods design  Poster presentation- project was in Phase 1 of the process (no completed version found in databases searched) | | - ED in Roskilde, Denmark - Wait times of (n=220) patients were analyzed | | - Phase 1: Quantitative data collection regarding wait times for category 3 and 4 patients in the ED - Phase 2: Implementation of a communication guide for one month, with nurses and doctors being interviewed over 7 days at the end of the implementation period to obtain their insights. - Poster presentation | | - A lack of initial dialogue between nurses and doctors about the necessary treatment and the plan of care for the patient increases wait times. - By enhancing teamwork and optimizing this initial communication to establish a shared care plan, the efficiency of ED patient care can be significantly improved. | |
| Brixey et al. (2007), United States) | | To explore the role of ED RNs and MDs as initiators of interruptions. | | Grounded Theory | | - Level One Trauma Center in the Gulf Coast region of the United States. - Convenience sample of **5** attending trauma physicians and **8** ED RNs were shadowed. | | - Two observers shadowed participants and documented minute-by-minute observations using an electronic semi-structured field note form. - Participants were observed for anywhere from two to twelve hours. - Field notes were analyzed and coded by both observers. | | - ED MDs and RNs tend to use face-to-face communication and telephone calls to initiate interruptions. - ED MDs only initiate 2.1 interruptions per hour but receive far more than this hourly. - ED RNs initiate more interruptions than MDs, likely because of their role in patient care coordination. - ED RNs receive far more interruptions than they initiate. - A taxonomy was developed to understand the dynamics of interruptions, categorizing them according to role (e.g., “Initiator”, “Recipient”, and subcategories such as “Delayed” and “Blocked”) - Cell phones and mobile technologies are changing how interruptions occur and this requires further investigation. | |
| Brixey et al. (2008), United States | | To monitor, document, and contextualize interruptions and activities of registered nurses (RNs) and physicians working in a Level One Trauma Center. | | Ethnography/Case study | | - Level One Trauma Center in the Gulf Coast region of the United States. - 5 attending ED physicians and 8ED RNs were shadowed. | | - Two observers shadowed participants for anywhere from two to twelve hours, keeping semi-structured notes throughout. - Time-stamped observations were analyzed using constant and line-by-line comparison and were then coded. - Taxonomy of interruption, interruption medium, impact of organizational design/artifact, and resumption of original activity were recorded. | | - 12.43% of ED physician activities and 4.11% of ED RN activities were interrupted. - Face-to-face interruptions occurred most often, with telephone calls, pagers ringing and self-interruptions being other interruption mediums. - ED Physicians were interrupted by phone calls more often than RNs. - Organizational design and supplies/equipment not being available in the immediate workspace contributed to interruptions. - In most cases, ED RNs and physicians completed only one interrupting activity before returning to their original task. - Emerging new technologies, including mobile devices, cause ED physicians and RNs, to be constantly and instantly susceptible to interruptions. - ED RNs and physicians should weigh the importance of an interruption against its negative impact on smooth and efficient workflow. | |
| Brixey et al. (2010), United States | | To examine ED registered nurses (RNs) and medical doctors (MDs) as initiators and recipients of interruptions. | | Grounded theory | | - Trauma section of a Level One trauma center academic hospital in the Gulf Coast region of the United States - Convenience sample of 8 male RNs, 6 female RNs, and 5 MDs working during shifts with high interruption rates. - Voluntary participation and informed consent obtained | | - Instrumental case study - Shadowing method used by two observers to collect observations about interruptions that were initiated and received by participants. - Shadowing occurred in sessions ranging from two to twelve hours in length. - Triangulation of quantitative and qualitative methods was used for data analysis. - Recorded noted categorized into codes based on event type and a developed role-based taxonomy | | - ED RNs and MDs were more often the recipients of interruptions than they were the initiators, - RNs are more likely to initiate interruptions than MDs. Of the observed interruptions, RNs initiated 36.16%. - 25.19% of ED MDs activities were interrupted, averaging 20.58 interruptions received per hour. MDs only initiated 2.1 interruptions hourly. - ED RNs experienced interruptions during 16.45% of all activities, averaging 11.65 interruptions per hour. - ED RNs and MDs usually initiated interruptions face-to-face, with telephone calls being the second most common communication medium used. - Both ED RNs and MDs more often resumed tasks than abandoning them after an interruption. - The impact of mobile technology on interruptions in the ED requires further research. | |
| Coiera et al. (2002), Australia | | To assess the communication demands on clinical staff in the ED and to outline patterns of both formal and informal communication events. | | Qualitative observational design | | - One rural ED and one urban ED in New South Wales between June and July of 1999. - Participants included 6 ED nurses and 6 ED physicians | | - One of two researchers shadowed participants for 1.5-2-hour time blocks during the morning, afternoon, and evening shifts. - Participants were given microphones to wear on their lapel and a transmitter to carry with them. - Participants had to obtain patients’ consent to keep the microphone on while providing/discussing care. - The researcher observed communication events from a distance. - Field notes were kept regarding event duration and parties involved. - Lapel microphone recordings were transcribed. - Communication events were coded for channel, other party, and purpose. | | - A total of 35 hours and 13 minutes’ worth of communication was observed. - ED nurses and physicians face a high communication load, with an average of 36.5 communication events per hour. - Nearly a third of these communication events are interruptions (11.5 interruptions per hour on average). - An average of 10% of communication events for both groups occurs while the individual is already completing another task. - ED physicians experience more multitasking alongside communication than nurses (14.6% vs 6.7%, respectively) - 89% of communication events used synchronous channels, with face-to-face conversation being the preferred channel, accounting for 82% of events. - Almost 90% of communication events were informal in nature. - Most communication events (94.8%) were for the purpose of information exchange between clinical staff. - The authors advocate for communication training for ED nurses and providers to increase awareness regarding the negative impact of interruptions. | |
| Cunningham (2021), South Africa | | To explore and better understand the interactions and communication between doctors and nurses in the emergency department. | | Commentary | | Not applicable to this type of literature source. | | Not applicable to this type of literature source. | | - ED nurses and providers face a high cognitive load and communicate frequently in a fast-paced, dynamic setting. - The power differential between ED nurses and providers needs to be addressed as it may negatively impact effective communication. - Hospital structures and organizational norms may perpetuate professional separatism and a culture of hierarchy, contributing to a lack of self-perceived agency by nurses and poor interprofessional relationships. - If nurses believe they have less agency than providers, they feel powerless and they are less likely to voice their concerns or share their inputs as they do not believe that these will be acknowledged or viewed as important. - A shared vision and integrated communication channels between nurses and providers are essential to collaboration and effective interprofessional information sharing. | |
| Daheshi et al. (2023), Saudi Arabia | | To evaluate nurses’ views on the quality of communication between physicians and nurses, along with related factors, in the emergency departments of selected government hospitals in Saudi Arabia. | | Cross-sectional study | | - Eight EDs of government hospitals in Hail and Jazan provinces, Saudi Arabia - A convenience sample of (n=250) staff nurses working at these eight EDs was used. | | - Data collected between January and June 2022 using a two-part self-administered questionnaire. - Part 1 assessed demographics; Part 2 focused on nurse-physician communication quality. - Data were analyzed using SPSS v. 24. - Descriptive statistics described participants’ characteristics, and the Kolmogorov-Smirnov test confirmed normal distribution (p > 0.05). - Independent sample t-tests and one-way ANOVA identified factors affecting nurses' perceptions of communication quality. Multiple linear regression determined independent predictors, with statistical significance set at p < 0.05. | | - Communication between ED nurses and physicians is considered subpar, with nurses giving it a mean score of 60.14 out of 90 across various domains. - Age, years of experience education level, and job position were positively correlated with nurses' views on nurse-physician communication quality. - Nurses over 30, those with diplomas, over 10 years of experience, and those in supervisory roles had better perceptions of communication quality. - There were no significant differences based on sex, marital status, nationality, or working hours (p > 0.05). Multiple linear regression indicated that none of these factors independently influenced nurses' perceptions of communication quality in emergency departments (p > 0.05). - Nurses reported high perceptions in the openness domain, indicating they feel comfortable collaborating with physicians and gain valuable knowledge through conversations with them, likely because they are familiar with physicians, they work closely with in the ED. - Encouraging nurses to provide input to physicians is vital for patient safety and can be achieved through strong management that promotes open communication among healthcare workers. - Joint training for nurses and physicians to ensure mutual appreciation of each specialty's contributions is suggested. | |
| Daouk-Öyry et al. (2017), United States | | To create a competency model for emergency physicians from nurses' perspectives, compare it with the Accreditation Council for Graduate Medical Education (ACGME) model, and identify unique competencies from the nurses' viewpoint. | | Comparative cross-sectional study | | - ED of a large academic hospital in the Middle East - ED RNs who had worked with the physician to be evaluated for at least 2 years and 2 shifts per month. - (n=36) total RN participants; 27 RNs evaluated 20 physicians in cycle 1; 33 RNs evaluated 21 physicians in cycle 2. | | - 13-item web-based questionnaires administered to ED RNs during biannual performance reviews - One of the items was open-ended and asked RNs to give feedback about physicians’ performance. - Content analysis was used to analyze the answers to the open-ended item | | - A nurse-led competency model for ED physicians was developed, consisting of eight core competencies. - “Communication skills” were identified as one of the eight core competencies.   RN’s Communication Suggestions for ED Physicians:   - Tone: Use a kind and respectful tone; Maintain a calm tone, particularly under stress and in the patient’s presence - Actively communicate: Use professional language, maintain eye contact, and actively listen. - Technical communication: Clearly communicate orders, care plans, and instructions to RNs and other staff. Follow up with both patients and staff to ensure understanding and adherence to the communication. | |
| Eppich (2015), United States | | To explore the factors contributing to communication breakdowns in high-risk healthcare settings, such as emergency medicine and pediatrics.  To better understand perceived barriers to voicing concerns among ED clinicians.  To identify strategies to foster a culture of psychological safety and effective communication to improve patient safety in the ED. | | Narrrative review | | Not applicable to this study design. | | Not applicable to this study design. | | - Communication breakdowns in high-risk settings, such as the ED and pediatrics pose a threat to patient safety. - Clinicians not feeling comfortable enough to provide their input or to voice questions/concerns is a large component of this issue. - Factors influencing clinicans’ hesitation to speak up include authority gradients, such as those between nurses and providers, previous experiences of disruptive or unprofessional behaviors, and the complexity of the healthcare socialization process. - Organizational change and management’s support is important for establishing an ED climate which fosters psychological safety. - Establishing this safety climate can help clinicians to find their voice and to feel supported to speak up . | |
| Fairbanks et al. (2007), Unites States | | To identify and describe intra- and inter-group communication patterns and links among ED clinician types. | | Prospective observational task analysis | | - The ED of a tertiary. university hospital that serves as the regional trauma center. - A convenience sample of 20 ED clinicians, 10 from the adult care section of the ED and 10 from the pediatric section. - Of the 10 clinicians observed in each area, 2 were attending physicians, 2 were registered nurses, and 2 were charge nurses. Remaining clinicians were resident physicians | | - The researcher tracked the mode, duration, location, partner, and any interruptions which occurred for each communication event on a paper data collection form. - For each measure, frequency percentage and duration percentage were calculated. - Link analysis graphs were used to help facilitate data interpretation. - Social interactions and interactions during direct patient care were excluded to ensure the focus remained on professionally related communication. | | - Communication is critical to key ED processes, and it is achieved through several means, including verbal, and written. - 39 hours and 12 minutes of communication were observed, during which participants experienced 1665 communication events. - RNs experienced the bulk of communication events, suggesting that they act as a hub for ED communication and are central to information sharing. - Face-to-face communication was the preferred means of communication and accounted for most events observed. - Most communication events took place in the nurse-physician station. - In the adult care area, communication events were briefer but more frequent than in the pediatric area. - Interruptions were more frequent in the adult care section than in the pediatric section. - Physicians faced more interruptions in comparison to bedside/charge nurses (6.9 per hour vs 0.5 and 3.9, respectively) | |
| Gharaveis et al. (2018), United States | | To explore whether increasing visibility in the ED improves collaborative communication and teamwork and reduces security concerns. | | Exploratory qualitative design | | - ED in Texas, United States - Of the (n=5) participants interviewed, three were nurses and two were physicians - Inclusion criteria were three years of work experience, with at least one year at the current facility, and work experience at other EDs. | | - Four hours of qualitative observation (half in the morning and half in the afternoon) during ED peak hours to assess visibility. - Field notes coded. - Five semi-structured, 30-minute in-person interviews with the researchers - Interviews recorded and transcribed, with transcripts going through verification. - Two rounds of coding for field noted and interview notes followed by thematic analysis | | - Where ED nurse-provider communication occurs depends on patient census; with high patient loads, nurses spend more time in patient rooms and hallways, communicating with providers there, whereas during lower census more communication occurs at the central workstation. - When trauma patients are brought to ED, a substantial amount of nurse-provider communication occurs at the bedside. - Participants felt visibility is a significant environmental element in the ED and felt high visibility improves communication, teamwork, and security. - Physicians valued staff-to-staff visibility more than ED nurses. - Better visibility reduces distractions during communication and improves concentration for medical staff. - Face-to-face communication, which is supported by increasing visibility, remains the gold standard according to participants for efficient and effective ED communication despite technology-based alternatives. - Nurses mentioned high levels of noise pose a barrier to effective communication in the ED, and suggested interventions targeting background noise reduction would be beneficial. | |
| Gharaveis et al., (2020a). United States | | To investigate how visibility affects teamwork in the ED. | | Mixed-methods design (exploratory qualitative; relational quantitative) | | - Four EDs within the same hospital system in Texas, United States - A convenience sample of (n=112) ED staff (28 males and 82 females; 10 physicians and 100 nurses) equally distributed across the four EDs, completed the teamwork survey - Interviews were conducted with (n=12) staff from each ED, with a minimum of one physician and two bedside RNs interviewed per site. - Inclusion criteria were three years of work experience, with at least one year at the current facility, and work experience at other EDs. | | - Depthmap 10 software used to measure ED visibility through morphology plans. - 12 hours of non-participant field observations were conducted during peak ED hours without recording. - A survey, field observations, and one-on-one interviews assessed teamwork. - Confounding variables included lighting, noise, number of annual visits, and staff’s work experience. - A light meter and an acoustic meter were used to measure lighting and sound. - Data about number of annual visits was obtained from ED management. - Job experience data was evaluated in the survey’s demographics section. | | - ED nurses and providers prefer face-to face communication. - Physicians value staff-to-staff visibility more, whereas nurses value staff-patient visibility. - Background surrounding workstations is a barrier to ED nurse-provider communication. - Survey responses and visibility measurements indicated that lower visibility may be associated with decreased teamwork and communication. - ED designs should include high-visibility layouts to optimize teamwork and communication. | |
| Gharaveis et al. (2020b), United States | | To explore the nature and extent of the relationship between visibility and collaborative communication in the EDs of four community hospitals | | Relational quantitative design | | - 4 EDs within a community hospital in Texas, United States - Convenience sampling yielded (n=109) volunteers for the survey portion of this study (99 nurses and 10 physicians). | | - Computerized floor plan analysis with measurements in Depthmap 10 software was used to assess general visibility (visual connectivity between different points in the ED environment) - The targeted visibility of staffs’ locations as the focus of the author’s line of sight was measured through 48 hours of observation (12 hours per site) - Sight lines between staff areas were documented on average every 18 minutes. - Face-to-face communication frequency and duration was measured using a smartphone timer and documented in a spreadsheet. - The survey tool was pilot tested and then hard copies were distributed to measure perceived collaborative communication among nurses and physicians | | - Visibility is important as it supports successful communication and promotes better outcomes. - Nurses view visual connectivity with other nurses and providers as beneficial to the delivery of care. - The results of six different analyses suggest a significant positive correlation between visibility (targeted or general) and communication (perception or face-to-face) - A high-visibility environmental design is optimal for effective nurse-provider communication in ED. - Specific attention should be paid by designers to maximizing visibility of central areas in EDs, such as workstations | |
| Gilardi et al. (2023), Italy | | To explore the essential. Elements of collaborative teamwork in EDs that could affect information flow. | | Exploratory ethnographic design | | - Two EDs (pediatric and general) within a large hospital in northern Italy. - (n=26) interviews with a combination of ED physicians, bedside nurses, triage nurses, and department heads. | | - Working practices of both EDs observed for 48 hours over a six-week period. - Information flow was observed from the triage desk to the treatment area. - Field notes were used to record observations. - Interviews with ED team members were conducted at the end of each shift. - Field notes and interview responses were anlayzed in the context of the theoretical framework, Distributed Cognition theory. - Two discussion groups, one for each ED and both three hours in duration, were organized to validate the preliminary findings from researchers’ observations and interpretations of interview responses, | | - ED triage nurses communicate their initial assessment of patients, highlighting key details, to physicians via electronic charting, as they do not work in the same space. - ED triage nurses expressed frustration with how physicians often do not read their triage notes, and that they sometimes need to follow-up with face-to-face communication to ensure the information is received. - Some ED physicians felt that triagists were ‘invading their turf’ by highlighting information, and they weighed the information provided by the triagist based on their perceptions of how reliable that nurse is. - ED treatment room nurses serve as ‘memory keepers’ and ‘process organizers’. - As ‘memory keepers’, nurses monitored laboratory report statuses, managed open cases, traced patient re-evaluations, reminded physicians of patient locations and specialist schedules, and coordinated patient flow and clinical assessments, effectively becoming the central point for all supplementary data. - As process organizers, ED nurses supported physicians by highlighting crucial information and engaging in cross-monitoring to ensure accurate data entry, especially towards the end of shifts when errors were more likely. This cross-monitoring was not agreed upon through verbal exchange; nurses took on the role autonomously. - ED physicians can impede nurses in their role as process organizer through delegitimizing behaviours, leading to a perceived loss of power. - Hierarchal dynamics in the ED contribute to differences in cognitive load among nurses and providers, and maintaining traditional authority gradients leads to relational tensions. | |
| Hacker Teper et al. (2022), Canada | | To investigate barriers/facilitators to escalation of care in the ED; To identify tools which can help clinicians to escalate care in the ED setting | | Scoping review | | Not applicable to this type of literature source. | | - The Arksey & O’Malley framework was used. - Review completed in accordance with PRISMA-ScR checklist. - 3 databases searched: MEDLINE, EMBASE and CINAHL. - Qualitative content analysis used by two reviewers to extract data; a third reviewer resolved any discrepancies. - 4257 records were identified; after screening, 13 were included. | | - Effective interpersonal communication between ED nurses and physicians, particularly the charge nurse and the most responsible physician, is key for timely recognition of patient deterioration and escalation of care. - Using structured communication tools, such as SBAR (Situation, Background, Assessment, Recommendation), can help nurses to present the urgency of a patient situation when contacting the ED provider. - Inadequate ED staffing and high workloads can make it difficult for nurses to contact providers when care needs to be escalated, and for providers to respond in a timely manner. | |
| Hertzum (2011), Denmark | | To examine variations in how ED clinicians perceive and assess electronic whiteboards, comparing differences among staff groups and departments at two different time points. | | Quasi-experimental design | | - Two EDs in Region Sjaelland, Denmark, and a pediatric department in Denmark to serve as a comparison group. - Surveys were distributed to 187 individuals in in the first round and 130 individuals in the second round, with a 51% response rate (n=161 useful responses) - Respondents were mainly nurses, followed by physicians and secretaries (specific number of participants from each staff group not provided by the authors). | | - Both surveys were pilot tested - The first survey was administered after initial clinician training with whiteboards to staff from both EDs and the pediatric department. - This initial survey established differences in how ED and non-ED clinicians assess whiteboards differently. - The second survey was administered 8-9 months after whiteboard implementation only to ED clinicians. - Both surveys included 21 closed questions about whiteboard implementation - The first survey included 7 additional questions about clinicians’ satisfaction with the work environment and their general attitude toward technology. - The second survey included an additional 3 closed questions concerning how clinicians perceived the electronic whiteboards. - Data from closed questions was analyzed using non-parametric tests | | - Respondents reported a more positive user experience with the electronic white boards in comparison to the previously used dry-erase boards. - Clinicians found the overview of information provided on the electronic boards to be useful. - In comparison to nurses’ experiences, physicians found it easier and faster to locate information on the electronic whiteboards. - Nurses and providers found the whiteboards were more useful in the acute care section of the ED where patients’ length of stay is longer and there is a greater need for prolonged interprofessional team communication. - In the comparator group, the pediatric department, electronic whiteboards were perceived as less important. - The authors support previous studies which suggested central placement of electronic whiteboards in the clinical setting optimizes their functionality. | |
| Hettinger et al. (2019), United States | | To identify ED nurses’ and physicians’ communication needs, specifically what content should be communicated, by whom, and at what time based on unique clinical scenarios | | Descriptive qualitative design | | - Two EDs at tertiary, teaching hospitals within a not-for-profit, health network in an urban area of the United States - Nurses (n=9), ED attending physicians (n=8) and ED resident physicians (n=4) working in the two EDs were interviewed. | | - Semi-structured interviews and focus groups were conducted in hospital conference rooms by the main author and two other researchers. - The remaining researchers joined the interviews by video call. - Of the 6 sessions, the average duration was 90 minutes. - Researchers took notes, and audio recordings of the sessions were used to validate notes. - Concept maps were created and refined through a 6-step process with a resulting knowledge model that combined the concept maps. | | - Share assessment findings and the plan of care, particularly the disposition, with other team members as soon as possible to avoid delays. - Communicate outstanding tasks /remaining steps in the patient’s care to ensure holdups or changes are addressed in a timely manner. - Communicate with the provider to coordinate proactive testing and therapeutic interventions to reduce delays in care when door-to-provider time is lengthy. - Don’t assume another team member is already aware of a piece of information; communicate new information to ensure a shared understanding. - Notify providers in a timely manner of any abnormal vital signs or changes in patient status. - If a task needs to be completed immediately, use verbal communication in addition to electronic orders to avoid delay by the time the nurse can check the electronic health record. - Instead of interrupting, use asynchronous communication, when possible, for low-priority items. - Use communication strategies, such as team huddles, to allow for introductions so team members can be familiar with everyone’s role and level of experience. - Adapt communication to fit the physical layout of the ED, particularly if nurses and physicians do not share workspaces, as this limits physical proximity and reduces situational awareness. - Implement strategies that leverage experience level, irrespective of clinical role/position in the organization, to ensure experienced staff members who possess institutional knowledge have a voice | |
| Hou et al. (2021), China | | To explore if a relationship/correlation exists between nurses’ moral distress and ethical climate, the nursing practice environment and nurses’ perceptions of ethical climate and the practice environment. | | Descriptive cross-sectional design | | - Five hospitals in Taiyuan, Shanxi Province in mainland China - A convenience sample of (n=300) ED nurses - Participants had a minimum of one year of work experience and had to be over the age of 18. | | - The questionnaire used combined the Moral Distress Scale-Revised (MDS-R) and the Hospital Ethics Climate Survey (HECSS) - An informational video about the study and a cover letter with informed consent was included for participants with the questionnaire. - Participants were given 1 month to complete the questionnaires. - SPSSS was used for data analysis. | | - ED nurses in mainland China experienced a low level of moral distress. - The more positive nurses’ perceptions of ethical climate and the nursing practice environment in their ED, the lower their level of moral distress - Poor nurse-physician communication and collaboration significantly contributes to the moral distress of ED nurses. - The power imbalance between ED nurses and physicians leads to mistrust, lower autonomy, and a lack of respect perceived by nurses, which increases moral distress. - Because the ED is a dynamic, rapidly changing environment, effective communication is challenging, and this may compromise collaboration. - The hierarchy between ED nurses and physicians further hinders communication as it reduces nurses’ assertiveness and causes them to use obliging conflict management and communication styles, which contributes to moral distress. | |
| Källberg et al. (2017), Sweden | | To describe the experiences of ED clinicians concerning risks to patient safety. | | Phenomenological design | | - Two Swedish EDs; the larger of which was in an urban university hospital and the smaller of which was in a county hospital. - 20 participants (10 RNs and 10 physicians; 5 of each group from each of the EDs) | | - Telephone interviews were conducted by two professional interviewers using a semi-structured interview guide, which was pilot tested. - Interviews lasted between 12-57 minutes (mean duration of 30 minutes) and were audio recorded. - Recordings were transcribed. - Inductive content analysis was completed. - Transcripts were re-read to allow for meaning units to form; these were then condensed into codes which were then sorted categorically. - Discussion among researchers reached a consensus regarding analysis results. | | - RNs and physicians perceived 4 main areas which pose risks for patient safety in the ED: communication failure, lack of control, high workload, and organizational failure. - Participants view high workloads to be the main cause of communication failures, including a lack of information being shared, and flaws in information delivery/receipt. - RNs and physicians also voiced concerns about how communication failures in the ED can lead to medication errors, incorrect testing, and adverse patient outcomes. - ED staff also recognized that even when communication is effective, interruptions often occur, and they can contribute to information being lost - Based on the experiences shared by ED RNs and physicians, interruptions are more likely to result in communication breakdown and jeopardize patient safety during periods with high patient load | |
| Leonardsen et al. (2024), Norway | | To explore the experiences of ED nurses with digitalization. | | Qualitative interview design | | - A medium-sized, modern hospital in Norway. - Purposive sampling was used. - Eight nurses were interviewed (7 currently working in ED and 1 ICU nurse with extensive past ED experience) | | - Semi-structured interviews followed an interview guide developed by the research team. - Interview guide pilot tested by two ED nurses and one ICU nurse. - Interviews conducted virtually via Microsoft Teams and audio/video recorded. - Transcripts analyzed with an inductive and thematic analysis approach. - Codes developed and verified by all members or research team | | - All ED nurses interviewed felt that digitalization negatively impacted communication between staff and between staff and patients. This contrasts previous studies on the topic, which generally reported positive impacts of digitalization. - ED nurses reported most ED communication is now done through digital messaging, which creates physical distance and reduces the opportunity for interprofessional collaboration. - Participants voiced concerns about missing messages from providers if busy completing other tasks in the ED, which may cause the message not to be received in a timely manner. | |
| Luu et al. (2021), United States | | To evaluate the impact of implementing Epic Secure Chat, an EHR-based messaging system, for non-urgent communication between ED RNs and physicians | | Quasi-experimental design | | - A large ED within a teaching hospital in the United States where physicians have a separate work area from nurses. - 17 physicians completed the pre-survey (*n* = 17); 16 completed the post-survey (*n* = 16) | | - Guidelines were distributed to help guide ED nurses and physicians in their decision-making regarding what constitutes nonurgent communication. - RNs and physicians were told to use their best clinical judgement above all when deciding what information was nonurgent and could be secure messaged. - Electronic pre- and post-surveys were emailed to ED physicians using Google Forms - Pre-intervention surveys were completed May 2019; Post-intervention surveys were completed August 2019. - Surveys evaluated whether Epic Secure Chat impacted the ED workflow and physicians’ burnout level | | - Disruptions to physician workflow due to nonurgent calls decreased significantly (*p* = 0.029). - The mean number of nonurgent calls received hourly by ED physicians decreased from 58.8% to 6.3% post-intervention. - Physicians reported “often being appropriately alerted by RNs” 75% of the time in post-surveys, an increase from 29.4% pre-intervention. - Buy-in regarding this intervention from ED nurses increased due to reasonable physician response time for nonurgent issues via secure message. - Findings suggest that incorporating Epic Secure Chat effectively improves the efficiency of ED workflow and has the potential to reduce the risk of burnout in ED physicians. | |
| Martin & Ciurzynski (2015), United States | | To describe the findings of a performance-improvement project designed to enhance communication, teamwork, and nurse satisfaction in a pediatric emergency department through joint NP-RN assessments and standardized SBAR huddles. | | Performance improvement (PI) project | | - Pediatric ED in a Level 1 Trauma center in western New York State - (n=32)   participants, including g(n=30) full time and part time RNs and (n=2) board-certified NPs. Most participants identified as female and Caucasian. | | - The Huddle, SBAR, and Communication Observation Tool (HSCOT) was used to evaluate participants’ communication and teamwork, and to provide insight into the intervention’s feasibility. - A pre- and post-test Collaboration and Satisfaction About Care Decisions–PEDS ED (CSACD) survey was self-administered to evaluate participants’ satisfaction with NP-RN collaboration. | | - The CSACD instrument showed high reliability (Cronbach α = 0.97). - Joint evaluations by NPs and RNs occurred in 83% of patient encounters, with 86% involving a structured SBAR huddle. - Joint evaluations and structured SBAR huddles enhanced teamwork, leading to better collaboration and clarity in patient treatment plans. - RN satisfaction with joint evaluations was slightly higher than NPs, who desired more RN input. - Communication and teamwork scores improved post-intervention (communication: 5.68 to 6.59; teamwork: 5.47 to 6.46). - RN job satisfaction increased from 5.17 to 6.45. - In 86% of cases, NPs and RNs discussed the treatment plan and roles. | |
| O’Mara (1999), United States | | To explore the dynamics of communication between emergency physicians, patients, nurses, and medical colleagues, highlighting potential conflicts and offering strategies to resolve them, emphasizing the importance of appreciating diverse perspectives and adaptability for effective conflict management in emergency medicine | | Commentary | | Not applicable to this type of literature source | | Not applicable to this type of literature source | | - By not actively seeking out and appreciating ED nurses’ contributions to patient care, ED physicians create a barrier to effective communication and teamwork. This also reduces the likelihood of a collegial relationship between the ED physician and nurse. - If ED nurses feel their opinions are valued by physicians, they are more likely to provide input and are less likely to be frustrated, in turn boosting overall morale. Thus, challenging the hierarchal nature of healthcare is important. - Strategies to improve ED nurse-physician relation include communication and conflict resolution training. | |
| Parizad et al. (2017), Iran | | To investigate the experiences of negative workplace behaviours among Iranian ED nurses. | | Exploratory, qualitative design | | - Four Iranian EDs - (n=15) nurses (8 female, 7 male) who had faced unprofessional behaviours in the workplace | | - Face-to-face, in-person, semi structured interviews were conducted and audio-recorded. - Interview transcripts were analyzed using thematic analysis. | | - Conflict arises when ED providers and other colleagues do not respond when nurses ask for help. - ED nurses voice frustrations over not being able to find the provider and asking them repeatedly to go see a patient. - ED staff identified that an approach of blaming or shaming others for errors often contributes to conflict. - When physicians question the skills and expertise of ED nurses, nurses perceive this as demeaning, unprofessional, and disrespectful. - ED staff acknowledge that high workloads contribute to fatigue and stress which encourages verbal altercations and conflicts. - ED nurses feel undervalued, and the authors suggest organizational changes to recognize nurses’ valuable contributions and policies which deter unprofessional communication behaviours in the workplace. | |
| Pun et al. (2015), China | | To investigate ED nurses’ and doctors’ views of communication between clinicians and with patients, including barriers to success in the high-pressured ED setting. | | Ethnographic design | | - A large, trilingual ED in Hong Kong, China - 28 clinicians were included in the sample: 20 nurses (11 male, 9 female) and 8 physicians (all male) | | - Semi-structured interviews were conducted with clinicians (n=28). - Interviews were audio-recorded, and transcripts were generated and translated from recordings. - Transcripts were de-identified and Nvivo 9 software was used to code the data during thematic analysis. | | - The ED is a high-stress and fast-paced work environment where communication is prone to error due to its complexity and it being fragmented, rushed, and often interrupted. - Interdisciplinary communication is highly frequent in the ED, but it is often rapid due to time constraints. - Issues regarding the transfer of medical information in the ED include inconsistencies/omissions in health records and inadequacies during triage and handover. - Omissions often occur as one party presumes that the other is already aware of information. - Nurses voiced frustrations with not being updated by providers about patients’ plan of care, and consequently not being able to answer patients’ questions due to this lack of information. - Nurses and physicians who were of lower seniority in the ED disclosed anxiety related to asking more senior clinicians for clarification or verification, suggesting hospital hierarchy hinders communication. - The ED’s physical environment is not conducive to effective communication as it is loud and overcrowded. - The authors suggest all interdisciplinary communication should involve a spoken or written confirmation of receipt/understanding. | |
| Relias Learning (2011), United States | | To identify strategies that can be used to optimize ED physician-nurse communication at critical junctures in care. | | Qualitative action research | | - Atlanta, Georgia - Authors state Crico Strategies collaborated with provider participants. Exact sample size not specified. | | - Crico Strategies organized structured collaborative sessions to discuss and identify key communication issues that have a role in diagnostic errors within the ED. - Based on the identified vulnerabilities, provider participants developed communication strategies that can help to alleviate the risk of error. | | Recommendations developed during the collaborative sessions include:   - Physician-Nurse Huddles: allow effective communication and review of key information at critical moments during patient’s care - Triggers: incorporating system warnings that alert nurses and physicians of abnormal vital signs or worsening of a patient’s condition to allow a prompt response - Discharge Timeout: ED physician and nurse review all discharge information together to detect any errors/omissions. - Abnormal Vital Sign Reconciliation: the nurse should inform the provider of abnormalities in a patient’s discharge vitals before discharge to reduce the risk of adverse events.   Other Suggestions:   - Operational/Organizational Changes: to standardize processes and reduce unnecessary tasks that are unrelated to patient care. - Staff Education/Professional Development: simulation training followed by debriefing can help reinforce proposed communication strategies and better equip ED nurses and providers for success. | |
| Relias Learning (2018), United States | | To explore how communication issues between ED physicians and ED nurses can complicate medical malpractice cases, particularly when electronic medical records (EMRs) are used.  To highlight how a strong, unified defense is key in malpractice defence. | | Commentary | | Not applicable to this literature type. | | Not applicable to this literature type. | | - During malpractice cases, communication haps between ED physicians and ED nurses can lead to conflicting testimonies during depositions, which weaken the defense. - Delays in nursing documentation, challenges faced by providers in accessing nursing documentation, and a lack of EMR notifications about new nursing documentation being added are all contributing issues. - Maintaining clear communication between ED nurses and physicians is important, and it becomes particularly significant when protecting against legal challenges. | |
| Relias Media (2020), United States | | To explore the legal issues that defensive charting by ED nurses can cause for both nurses and ED providers; To provide suggestions for creating a ‘strong culture of safety’ in the ED. | | Commentary | | Not applicable to this type of literature source. | | Not applicable to this type of literature source. | | - Defensive charting by ED nurses poses an increased legal risk for all involved in the patient’s care.   Suggestions for ED Nurses:   - Directly communicate concerns to ED providers and use open communication to resolve disagreements. - Avoid statements like “Provider informed of changes,” as the provider may miss or be unaware of nursing notes in the electronic chart, leading to unresolved issues. - Use incident reports to report concerns about ED providers to risk management. - Familiarize yourself with the chain of command at your institution and use it to address patient care issues | |
| Schneider et al. (2019), Germany | | To differentiate between helpful and harmful interruptions of ED providers using patients’ perceptions of ED care as a quality measure. | | Cross-sectional observational study | | - Adult ED at a tertiary academic hospital in Munich, Germany. - Convenience sample of nurses and physicians - Of the observation sessions conducted (n= 160), 99 were with nurses and 61 were with physicians. - (n=1418) patients were surveyed. All patients who received ED care on data collection days were eligible. | | - Systematic observations were collected over 40 days in 90-minute sessions by a three-member team during ED hours with peak census. - The source, frequency, and content of interruptions was recorded. - A standardized survey was used to assess patient perceptions of overall care quality, organization within the ED and wait times. - Hierarchical linear models were used to evaluate associations between patients’ ratings and interruptions in the ED, controlling for daily workload demands. - Results of regression analysis were adjusted to account for multiple testing. - Analyses for ED nurses and physicians were computed separately. | | - ED providers were most interrupted by colleagues from their own profession (M- 2.55 interruptions hourly; SD=2.39), colleagues from other professions (M= 2.47, SD=2.13), or via telephone/beeper (M=1.79, SD=1.52). - Content of interruptions most frequently included information about parallel cases, care coordination activities and issues related to current cases, respectively. - There were role-related differences in interruptions between ED nurses and physicians. In comparison to physicians, nurses received more interruptions related to patient comfort issues. - Interruptions related to coordination among ED nurses were closely tied to how patients view wait times, which highlights the critical role that nurses play in care coordination and collaboration in the ED. - Interruptions that were not relevant to current cases were correlated to lower patient ratings of organization in the ED. - When designing healthcare systems, the benefits, and harms of interruptions on ED workflow and patient safety must be thoroughly considered. | |
| Suryanto et al. (2016), Australia | | To compare the attitudes of ED nurses and physicians towards nurse-provider collaboration. | | Comparative descriptive quantitative design | | - General hospital in Malang, Indonesia. - Convenience sample of (n-47) ED nurses and (n=24) ED physicians surveyed. - Participation was voluntary. - Inclusion criteria: (i) Indonesian (ii) holds Vocational School of Nursing degree for nurses (iii) general practitioner for physicians (iv) currently working in the ED for both disciplines | | - A modified version of the Jefferson Scale in Attitude towards Physician-Nurse Collaboration was used. - Two-part questionnaire consisted of a demographics section and a section of survey questions. - Anonymous surveys were distributed by nurse coordinators to nurses and returned in sealed envelopes. Physicians received the surveys during a ‘Medical Reports’ meeting and returned completed questionnaires to a sealed box in the common room. - Descriptive statistics, parametric, and non-parametric inferential statistics were used to analyze data | | - ED nurses have significantly more positive attitudes toward collaboration than ED physicians (p<.001). - Nurses scored significantly higher in three of the four measured domains: “physician dominance”, “nurse autonomy”, and “caring versus curing”. - Factors such as gender, age, and education did not significantly influence nurses’ or physicians’ attitudes toward collaboration. - Experience in the hospital’s ED was significantly linked to participants’ attitudes toward collaboration (p= .02). More ED experience was linked to more positive attitudes towards nurse-physician collaboration, potentially due to experienced nurse and physicians being more comfortable in their work environment. - Organizations should create opportunities for interprofessional collaboration, such as through training programmes and mentorship opportunities. | |
| Tindle et al. (2020), United States | | To evidence-based data for emergency department designs by examining how different nurse station layouts (centralized vs. decentralized) affect nurse interactions and actions throughout a shift. | | Directly observed task performance time study and analysis. | | - ED of a major teaching hospital in the United States, split into two sides (A side with a centralized nursing station and B side with five nursing stations and a centralized physician workstation - (n=16) nurses working 12-hour day shifts were selected by simple randomization of patient room assignments | | - A task performance and direct observation time study utilized a novel tablet-based digital mapping tool created with the ArcGIS cloud-based platform. This tool accurately collected and mapped data on provider actions and interactions within the emergency department throughout an entire nursing shift. | | - There is a statistically significant difference in the duration of nurse-physician interactions between the ED designs on A side and B side (centralized vs decentralized). - In the centralized design, nurse-physician interactions totaled 14 minutes and 38 seconds compared with 30 minutes and 11 seconds in the decentralized design (*t.=* 1.31, *p*= 0.02) - More nurse-physician interactions took place inside the patient’s room in the decentralized design. Including the patient can allow for shared decision-making between the nurse, physician, and patient. | |
| Valentino et al. (2020), United States | | To improve the process used to alert providers of abnormal vital signs in the pediatric ED. | | Mixed-methods design | | - The ED of University of Chicago Comer Children’s Hospital (Level I Trauma Center) - 308 (*n* = 308) pediatric patients (2 months of age-18 years of age) with abnormal vital signs (156 pre-intervention; 152 post-intervention) - Most children were of African American descent. | | - A best practice (BPA) advisory was implemented over a two-month period to alert clinicians of abnormal vital signs through the electronic health record (EHR). - Education regarding the need for change was provided before implementation. - The BPA prompted clinicians to consider if there was a need to re-evaluate vital sings prior to discharge and/or to notify the provider. - The BPA was pilot tested with advanced nursing providers and registered nurses (RNs) working in the ED. - A system usability scale (SUS) survey was administered after the pilot to assess APRN and RN’s perceptions of the BPA. - Round-table discussions with participants provided qualitative data. - Chart review was used to evaluate effectiveness of BPA in achieving the desired outcome. | | - ED RNs are responsible for effective, timely communication with ED providers about abnormal assessment findings. - Pre-intervention, ED RNs did not consistently communicate abnormal vital signs to the provider, as this was done at their discretion. - The BPA improved the frequency of provider notification regarding abnormal vital signs and facilitated appropriate clinical decision making, including re-evaluation prior to discharge. - The BPA reduced the percentage of patients discharged from the ED with abnormal vital signs (15.6% vs 5.6% with abnormal temperatures; 10.9% vs 5.9% with abnormal respiratory rates; and 25% vs 11.9% with abnormal heart rates) - The authors suggest its implementation into all EHRs. | |
| Weaver et al. (2017), United States | | To determine if shared workstations for registered nurses and physicians, physician assistants, and nurse practitioners in the ED improve teamwork and communication. | | Prospective pre-post survey design | | - The ED of a university hospital in Dallas, Texas with 27 ED bays and separate workstations for nurses and providers - Relocation to a new location was planned for 3 months from the study’s commencement. - The new ED would increase in size to 40 bays and include 4 centrally located shared workstations for nurses and providers. - Study participants included medical doctors, advance-practice providers, and registered nurses. - There were 47 respondents to the pre-survey (n=47) and all but one of those respondents completed the post-survey (n=46) | | - Team STEPPS T-TPQ questionnaire was administered 3 months pre-relocation and 3 months post-relocation to 47 clinicians, including ED nurses and providers. | | - Shared workstations for nurses and provides positively impacted communication among team members according to survey responses. - The communication dimension saw the most significant improvements, indicating that shared nurse-provider workstations improved timeliness and clarity of communication, and allowed for efficient verification and clarification. - Situation monitoring scores increased, demonstrating that shared workstations improved the team’s ability to anticipate needs, exchange valuable patient information, and to modify team operations to avoid a negative outcome. - Shared workstations have the potential to improve ED nurse-provider communication and should be considered when designing/remodeling ED layouts. | |
| Williamson & Kives (1991), United States | | To evaluate the roles and relationships among ED nurses and physicians, to refine the definition of collaboration, and to establish guidelines for strengthening collaborative relationships in the ED. | | Commentary | | Not applicable to this type of literature source. | | Not applicable to this type of literature source. | | - In their relationships, ED nurses prioritize respect, trust, and open communication, whereas physicians see nurses’ cooperation and competence as crucial factors. - A collaborative practice model allows for nurses to be well-informed of patients’ plans of care and results in fewer phone calls, pages, and interruptions to physicians. - Physicians may feel threatened and have negative attitudes towards collaborative communication about a patient’s plan of care. - When nurses feel they cannot safely provide direct input to physicians and instead need to present suggestions as if they were the physician’s own idea to avoid conflict, this mimics how ED physicians approach consults with private physicians in the middle of the night. - A joint-practice committee or a nurse-provider forum can allow for interprofessional discussion and planning | |

**Concept Maps**

**Figure 1C**

**
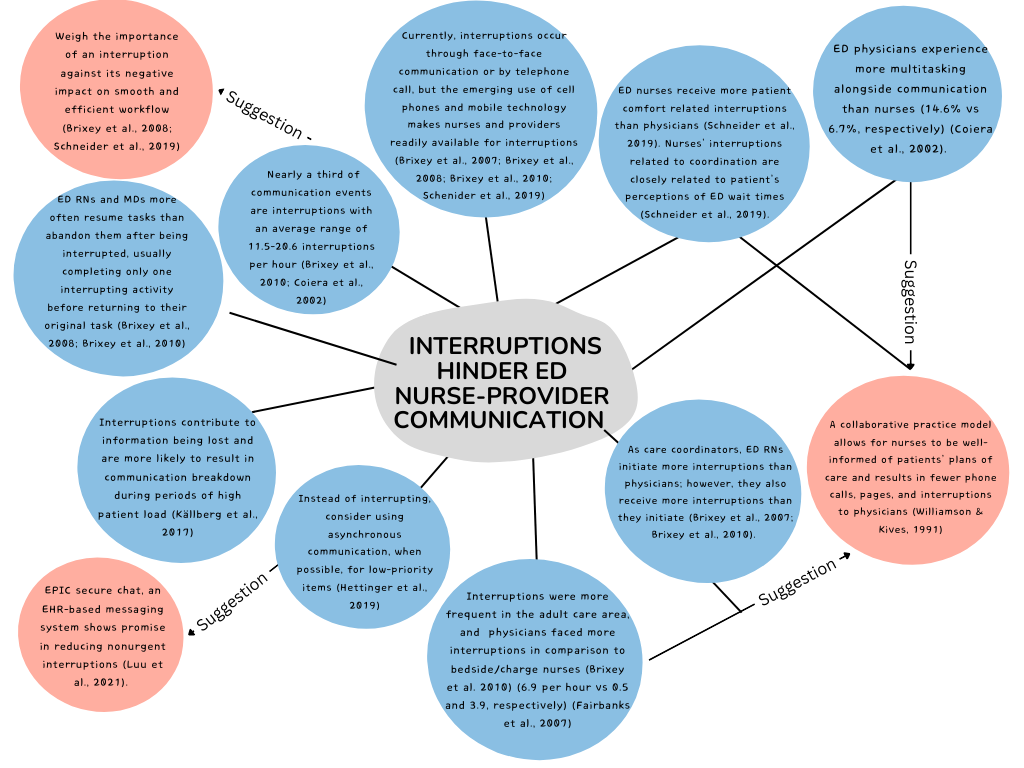
***Interruptions: A Barrier to ED Nurse-Provider Communication*

**Figure 2C**

**
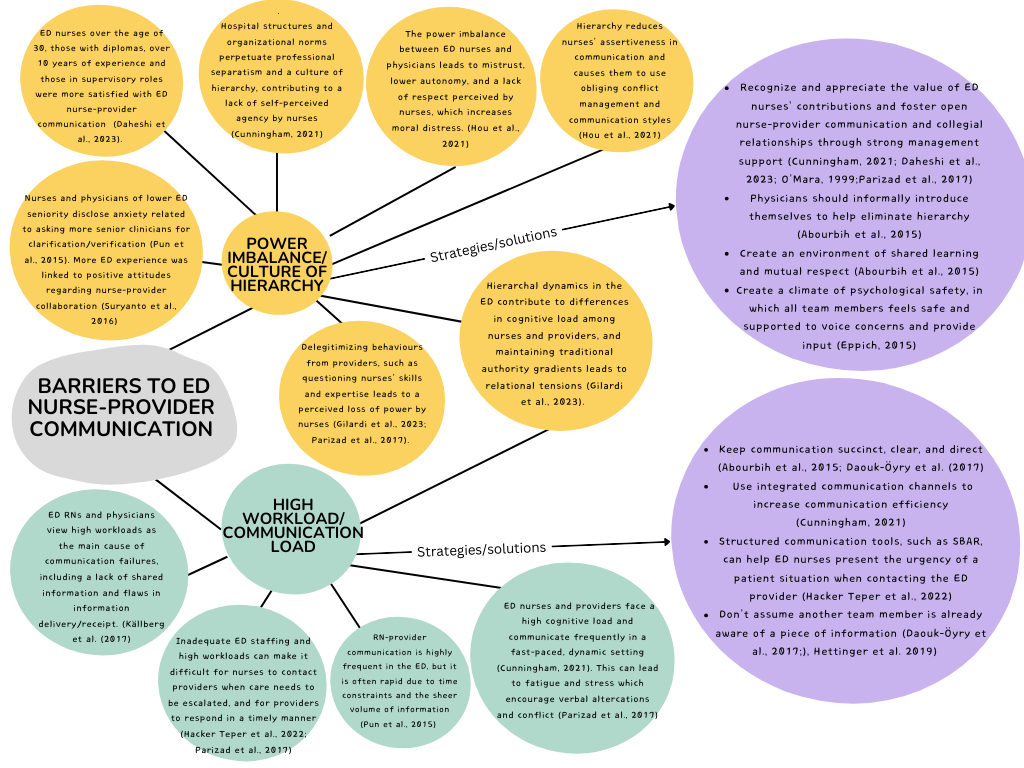
***Other Barriers to ED Nurse-Provider Communication*

**Figure 3C**

*Facilitators to ED Nurse-Provider Communication
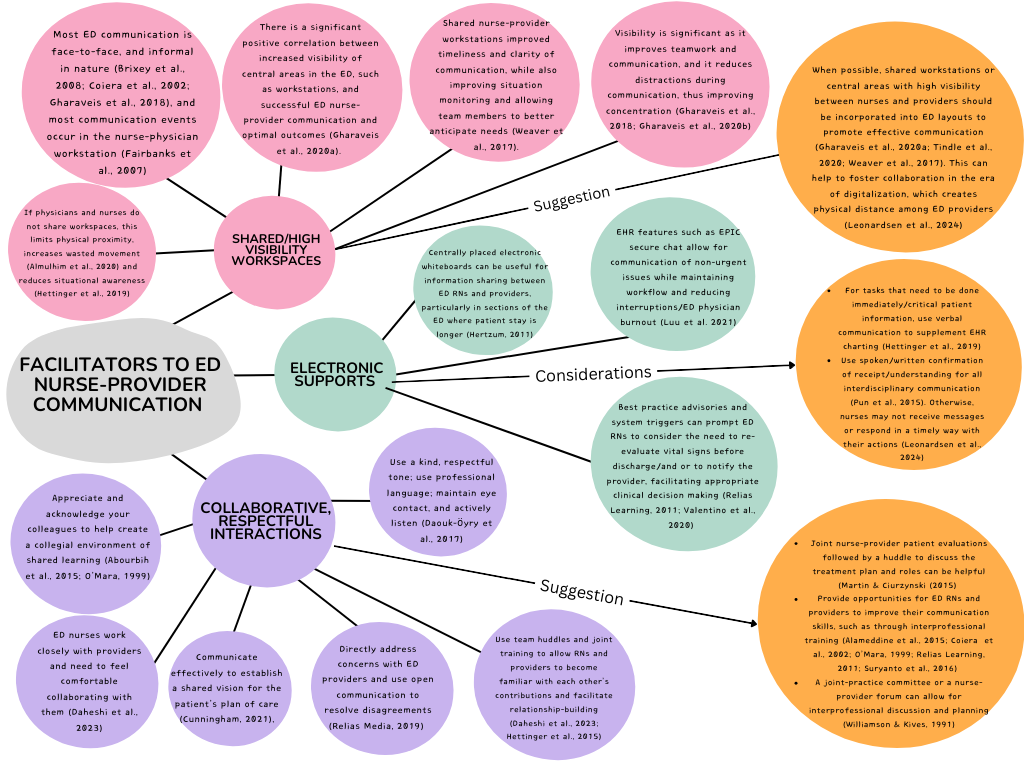
*

**Figure 4C**

*ED Nurse-Provider Communication: Valued Content* **
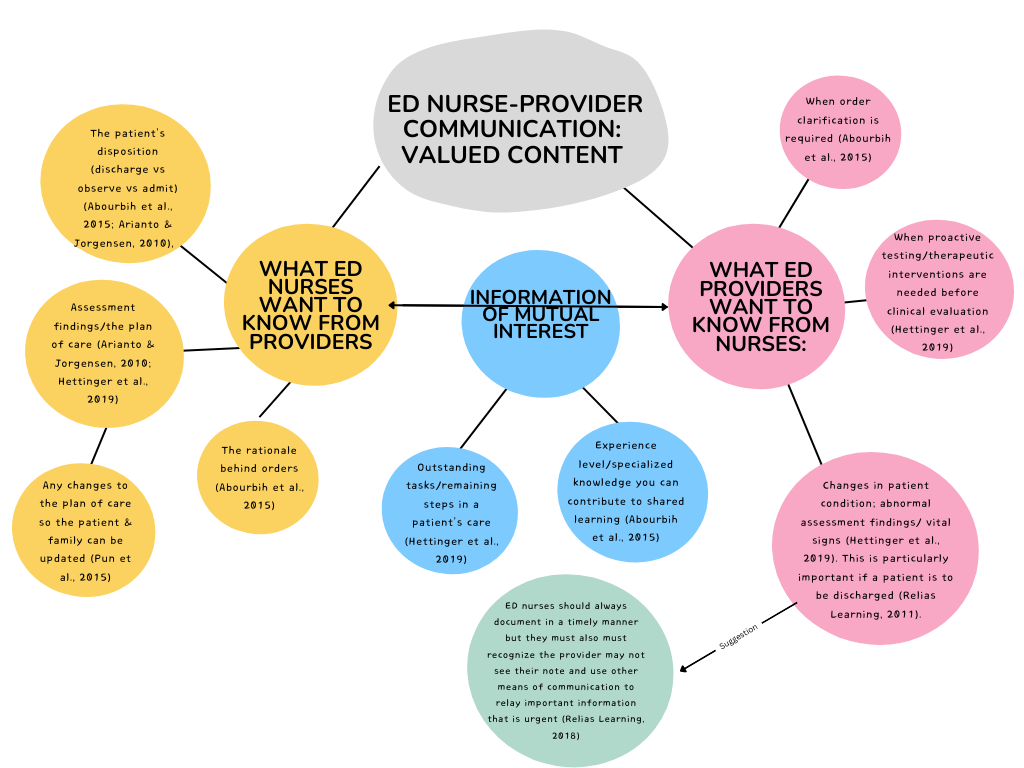
**

**Appendix C**

**Concept Maps**

**
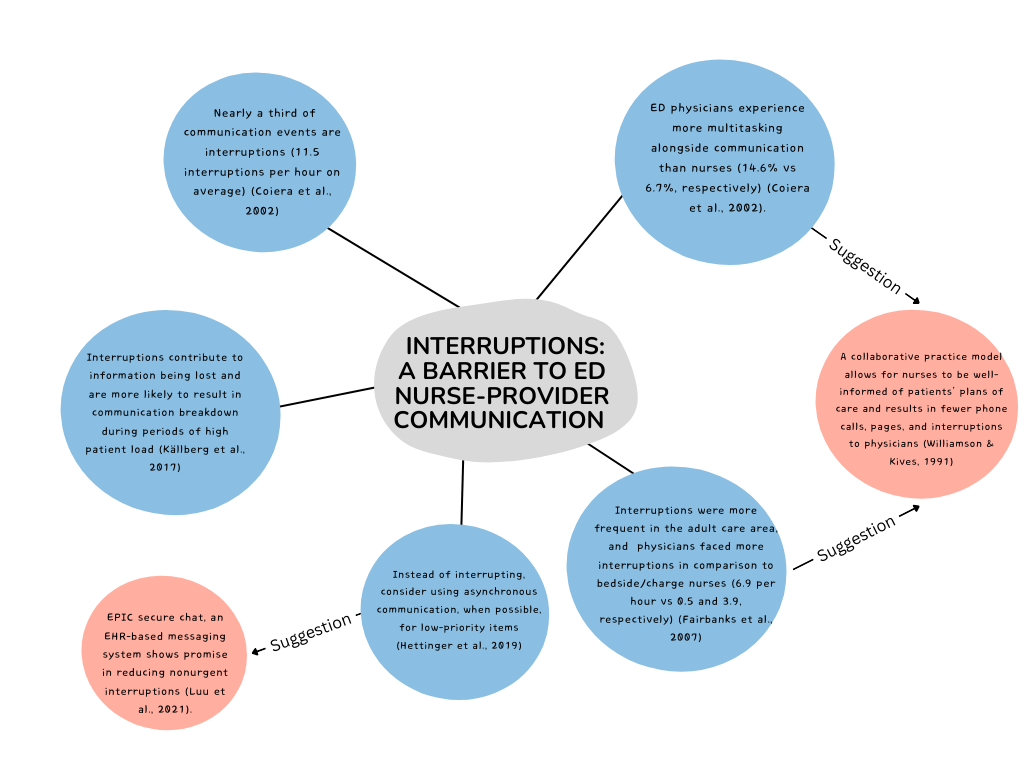
Figure 1C: Interruptions: A Barrier to ED Nurse-Provider Communication**

**Figure 2C: Other Barriers to ED Nurse-Provider Communication**

**
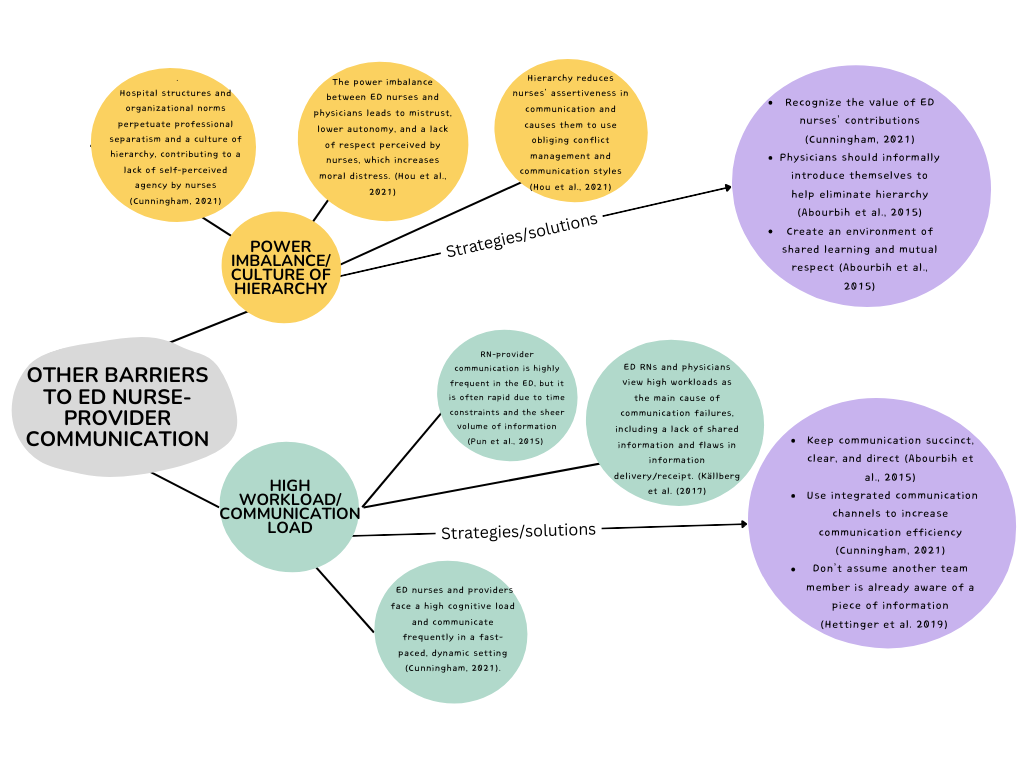
**

**Figure 3C: Facilitators to ED Nurse-Provider Communication**
